# Prevention approaches aimed at men who commit violence against women: a scoping review

**DOI:** 10.1590/1980-220X-REEUSP-2025-0036en

**Published:** 2025-07-07

**Authors:** Victoria Leslyê Rocha Gutmann, Gisele Cristina Manfrini, Tereza Maria Mendes Diniz de Andrade Barroso, Camila Daiane Silva, Sheila Rubia Lindner, Sandra Mara Corrêa

**Affiliations:** 1 Universidade Federal de Santa Catarina, Programa de Pós-Graduação em Enfermagem, Florianópolis, SC, Brazil.; 2 Universidade Federal de Santa Catarina, Departamento de Enfermagem, Florianópolis, SC, Brazil.; 3 Escola Superior de Enfermagem de Coimbra, Coimbra, Portugal.; 4 Universidade Federal do Rio Grande, Escola de Enfermagem, Rio Grande, RS, Brazil.; 5 Universidade Federal de Santa Catarina, Departamento de Saúde Pública, Florianópolis, SC, Brazil.

**Keywords:** Men, Primary Prevention, Secondary Prevention, Tertiary Prevention, Violence Against Women

## Abstract

**Objective:**

To map primary, secondary, and tertiary prevention approaches applied to men who commit violence against women.

**Method:**

Qualitative scoping review, according to JBI guidelines. The search was carried out from July to October 2022 in the MEDLINE, Embase, CINAHL, PsycInfo, Scopus, Web of Science, LILACS, BDENF, IndexPsi, SciELO and Google Scholar databases, without restrictions regarding geographic area, publication date, and/or language. All studies were assessed for reliability and credibility, according to the ConQual Score.

**Results:**

Twenty-eight studies were selected, most of which had a secondary and tertiary level approach. Only two studies fell within primary prevention. The interventions were mostly carried out in groups. The interdisciplinary/interprofessional dynamic, rarely described, involved areas such as psychology, social assistance, and other fields of social sciences.

**Conclusion:**

The interventions, in general, followed the group approach, with the potential to be replicated in other services, including health services and for other audiences, as long as they have the theoretical and human support for this, expanding the actions to the primary level of prevention.

## INTRODUCTION

Violence against Women (VAW) involves a complex evolution that transcends different phases of life, becoming a social and global problem, visible daily in significant data on women who suffer and are killed for being women^(1)^. Globally, around 30% of girls and women aged 15 to 49 reported having experienced physical and/or sexual violence by an intimate partner at least once, with a higher prevalence in low-income countries. In Brazil, this rate is 23%^(2)^. In 2022 alone, the country recorded 51,407 cases of physical violence. The most common place where VAW occurred was the home, in more than 80% of cases, with men being the main perpetrators, in 86.6% of cases. As for cases of feminicide, a rate of 3.5 cases for every 100,000 women is estimated, in which, again, men, specifically boyfriends and husbands, were the main perpetrators^(3)^.

The damage caused by violence, when not fatal, ranges from physical, including injuries and wounds, to psychological, involving psycho-emotional impairment of women, such as low self-esteem and common mental disorders (depression, anxiety, sleep disorders, post-traumatic stress and suicidal ideation), as well as hyperactivity, attention and learning difficulties, inadequate eating patterns, and use and/or abuse of drugs and medications^(4)^. Such repercussions also affect the family and people close to women in situations of violence, as well as society as a whole, making it an increasingly present demand in health services. Therefore, VAW is a global concern and an important public health problem, requiring actions aimed at confronting it and, especially, preventing it^(5)^.

In this regard, prevention approaches are divided into primary, secondary, and tertiary levels. Primary prevention includes actions taken before violence occurs, to prevent the incidence and prevalence of cases, that is, initial perpetration or victimization. Secondary prevention, in turn, seeks to reduce violence that is already underway, identifying individuals and families at risk through the existence of situational crises that trigger violence and, thus, focuses on an immediate response. Finally, tertiary prevention is based on long-term responses, aiming to offer assistance after the event, avoiding recurrence^(6)^. Although necessary, secondary and tertiary responses alone are not capable of reducing, nor of preventing or fully intervening with the incidence of VAW. Primary approaches provide a broad and holistic perspective in dealing with relational and family problems^(1)^.

Thus, efforts have been sought to go beyond secondary and tertiary prevention efforts to allow greater opportunities for primary prevention. It is believed that a healthy relationship structure offers ample opportunities for violence prevention, whether by fostering bonding between child and caregiver, encouraging mentoring relationships between young people and adults, or providing financial resources to reduce family economic stress^(7)^. This last topic, especially, has been associated with women remaining in situations of violence due to financial dependence. Such dependence occurs when the partner is the provider or exercises control over the family income. Furthermore, precarious employment and salary scenarios also contribute to women’s increased vulnerability^(5)^.

In addition to individual, psychological, and relational variables, men, specifically, are the result of a sociocultural construction of gender that normalizes and naturalizes male power over female power, based on patriarchal precepts that often bring them closer to so-called virile and, consequently, violent acts. This fact is expressed in situations in which men, upon perceiving their masculine identity as being threatened, may resort to violence^(8)^. Therefore, violence prevention must also consider the transformation and deconstruction of crystallized ways of thinking, acting, and feeling of hegemonic masculinity, as well as its social and affective function in relationships with women and children^(9)^. In other words, overcoming violence requires the construction of alternative ways of being a man, towards harmonious, equitable and non-violent marital relationships^(10)^. However, there are still few studies involving male perpetrators of violence, especially with a preventive nature and/or aimed at measuring the effectiveness of approaches involving this group^(11)^.

The World Health Organization recognizes VAW as a serious violation of human rights and an obstacle to achieving the Sustainable Development Goals (SDGs) and, therefore, has encouraged work and studies with men to promote gender equity and equality^(10)^. As for the SDGs, 5 and 16 stand out, which concern, respectively, gender equality to combat inequalities, including violence against women, and the promotion of peaceful societies. Specifically, SDG 5, which deals with achieving gender equality and women’s empowerment, is characterized as a historic commitment to confronting, preventing, and eliminating all forms of gender-based violence, given the persistence and adverse effects of this problem^(12)^.

Thus, to verify the body of evidence regarding approaches to preventing VAW, the need arose to conduct a literature review, specifically a scoping review, to provide an overview of the actions and strategies aimed at primary, secondary, and tertiary prevention of VAW, especially aimed at men who are perpetrators of violence. Thus, the objective of this study was to map primary, secondary, and tertiary prevention approaches applied to men who commit violence against women.

## METHOD

### Design of Study

This is a scoping review, carried out in accordance with the methodological guidelines of *Joana Briggs Institute* (JBI), following the steps: identification of the research question and objective; search strategy; selection of studies, according to pre- established criteria; mapping and extraction of data; analysis of evidence; and summarized presentation of results. This methodology seeks to provide an overview or map of the evidence and possible knowledge gaps, as well as explore the extent of the literature on a given topic, paving the way for future research with more specific targeting^(13)^.

The recommendations of the *Preferred Reporting Items for Systematic Reviews and Meta-Analyses* (PRISMA-ScR), which includes the use of a checklist for the preparation and reporting of the methodological steps of the protocol for this review^(13)^ were also considered. The review was registered on the platform *Open Science Framework*, with DOI identification: http://dx.doi.org/10.17605/OSF.IO/XGVA5.

### Selection criteria

To define the study question, the acronym PCC was adopted, where P - Population = men who commit violence against women; C - Concept = primary, secondary, and tertiary prevention approaches; and C - Context = violence against women. Thus, the following question was formulated: “What primary, secondary, and tertiary prevention approaches are applied to men who commit violence against women?”.

Based on this, the following inclusion criteria were defined: original research articles, of the qualitative type, dealing with primary, secondary, and/or tertiary prevention approaches aimed at men who are perpetrators of violence, of any nationality, race, or color. No limits were set on geographic area, publication date and/or language, except for full-text versions exclusively in Mandarin. The exclusion criteria applied were theses, dissertations, and duplicate or incomplete articles, and/or those that did not correspond to the research theme.

The search was carried out in 11 indexed databases and in the gray literature, namely: *PubMed*/*MEDLINE*, *Embase*, *CINAHL*, *PsycInfo*, *Scopus*, *Web of Science*, *LILACS*, *BDENF*, *IndexPsi*, *SciELO,* and *Google Scholar*. As a complement to the search strategy, the textual references of the studies selected for full reading were consulted, which could be included if they met the inclusion criteria and the question and objective of the study.

It should be noted that the search syntax was adapted for each database, in accordance with its format. The words contained in the titles and abstracts of relevant articles and the indexing terms used to describe the articles were analyzed by two authors and a librarian, to compose the strategy, in accordance with each PCC item, selecting a set of descriptors available in the Health Sciences Descriptors (DeCS), in *Medical Subject Headings* (MeSH), in addition to keywords, in Portuguese, English, and Spanish. The search strategy was developed by combining DeCS terms, *MeSH,* and keywords, with the application of the Boolean operators E/*AND* OU/*OR*. The search strategies and the association of descriptors with Boolean operators, for each database, can be seen in Chart 1.

**Chart 1:** Search strategy in the databases – Florianópolis, SC, Brazil, 2025.

Databases	Search strategy
*Pubmed/MEDLINE*	((“Violence Against Women“ OR “Crimes against Women“ OR “Gender-Based Violence“[Mesh] OR “Gender-Based Violence“ OR “Domestic Violence“[Mesh] OR “Domestic Violence“ OR “Intimate Partner Violence“[Mesh] OR “Intimate Partner Violence“ OR “Dating Violence“ OR “Intimate Partner Abuse“) AND (“Primary Prevention“[Mesh] OR “Primary Prevention“ OR “Prevention“ OR “Preventive Health Services“[Mesh] OR “Preventive Health Services“ OR Preventive* OR “Secondary Prevention“[Mesh] OR “Secondary Prevention“ OR “Tertiary Prevention“[Mesh] OR “Tertiary Prevention“) AND (“Men“[Mesh] OR “Men“[Title] OR “Man“[Title] OR “Male“[Title]))
*Embase*	((“Violence Against Women“ OR “Crimes against Women“ OR “Gender-Based Violence“ OR “Domestic Violence“ OR “Intimate Partner Violence“ OR “Dating Violence“ OR “Intimate Partner Abuse“) AND (“Primary Prevention“ OR “Prevention“ OR “Preventive Health Services“ OR Preventive* OR “Secondary Prevention“ OR “Tertiary Prevention“) AND (“Men“/de OR “Men“:ti OR “Man“:ti OR “Male“:ti))
*CINAHL* *PsycInfo* *Scopus*	((“Violence Against Women“ OR “Crimes against Women“ OR “Gender-Based Violence“ OR “Domestic Violence“ OR “Intimate Partner Violence“ OR “Dating Violence“ OR “Intimate Partner Abuse“) AND (“Primary Prevention“ OR “Prevention“ OR “Preventive Health Services“ OR Preventive* OR “Secondary Prevention“ OR “Tertiary Prevention“) AND (“Men“ OR “Men“ OR “Man“ OR “Male“))
*Web of Science*	NOFT((“Violence Against Women“ OR “Crimes against Women“ OR “Gender-Based Violence“ OR “Domestic Violence“ OR “Intimate Partner Violence“ OR “Dating Violence“ OR “Intimate Partner Abuse“) AND (“Primary Prevention“ OR “Prevention“ OR “Preventive Health Services“ OR Preventive* OR “Secondary Prevention“ OR “Tertiary Prevention“) AND (“Men“ OR “Men“ OR “Man“ OR “Male“))
*LILACS* *BDENF* *IndexPsi* *SciELO*	((“Violência contra a Mulher““ OR “Crimes contra a Mulher“ OR “Crimes contra as Mulheres“ OR “Violência contra as Mulheres“ OR “Violência de Gênero“ OR “Violência Doméstica“ OR “Violência por Parceiro Íntimo“ OR “Violencia contra la Mujer“ OR “Crímenes contra la Mujer“ OR “Crímenes contra las Mujeres“ OR “Violencia contra las Mujeres“ OR “Violencia de Pareja“ OR “Violence Against Women“ OR “Crimes against Women“ OR “Gender-Based Violence“ OR “Domestic Violence“ OR “Intimate Partner Violence“ OR “Dating Violence“ OR “Intimate Partner Abuse“) AND (“Prevenção Primária“ OR “Prevenção“ OR “Serviços Preventivos de Saúde“ OR Preventiv* OR “Prevención Primaria“ OR “Prevención“ OR “Servicios Preventivos de Salud“ OR “Primary Prevention“ OR “Prevention“ OR “Preventive Health Services“ OR “Prevenção Secundária“ OR “Prevenção Terciária“ OR “Prevención Secundaria“ OR “Prevención Terciaria“ OR “Secondary Prevention“ OR “Tertiary Prevention“) AND (“Homens“ OR “Homem“ OR “masculino“ OR “Hombres“ OR “Hombre“ OR “Men“ OR “Man“ OR “Male“)
*Google Scholar*	((“Violência contra a Mulher“ OR “Violência de Gênero“ OR “Violência Doméstica“ OR “Violence Against Women“ OR “Gender-Based Violence“ OR “Domestic Violence“) AND (Prevenção OR Prevention) AND (“Homens“ OR “Homem“ OR “Men“ OR “Man“))

### Data Collection

Data collection was carried out between July and October 2022, so that all studies retrieved from the databases and the first hundred from the gray literature were exported to the reference manager *EndNote Web*®, free version, in which duplicate results have been removed. Afterwards, to verify the correspondence of the articles to the research question, the reading of the titles, abstract, and descriptors was carried out on the platform *Rayyan Web*®.

**Figure 1:**
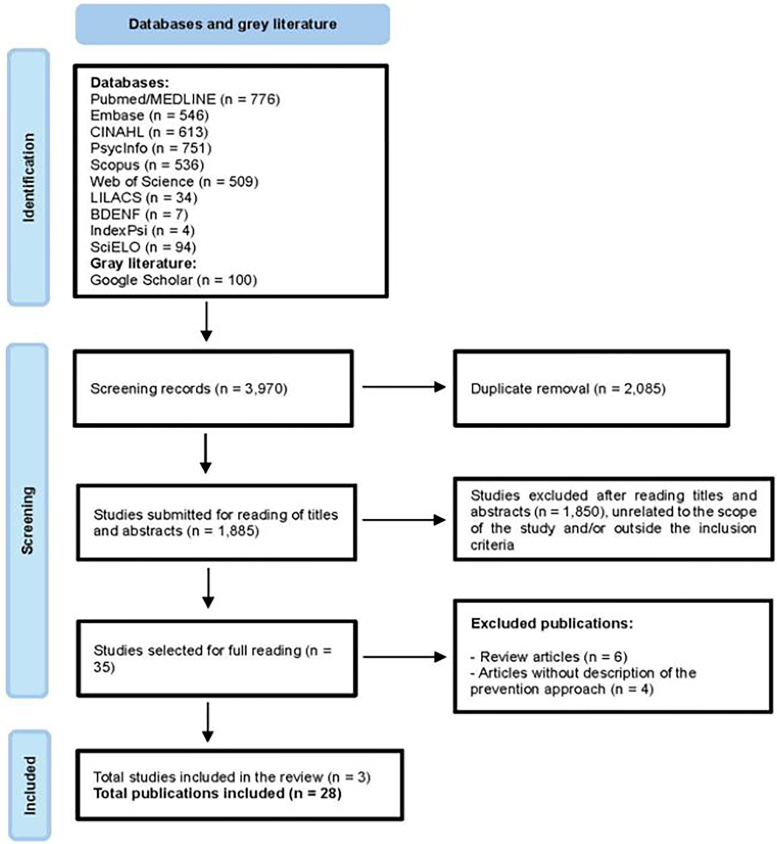
Flowchart of the selection process for included articles.

The double-blind method was followed and inclusion or exclusion occurred initially by the title, followed by the abstract, and finally, by reading the full text. After confirming the inclusion of the article, the reviewers also searched for potentially useful studies in their references. In case of disagreement, the possibility of consulting a third author was provided, which was not necessary during the process. The results of the search and the process of identifying and selecting studies, as well as the reasons for exclusion, were recorded and reported in the PRISMA-ScR flowchart, as shown in Figure 1.

### Data Analysis and Treatment

After reading the included articles in full, the reviewers highlighted and summarized the information that answered the question and objective of the review. Thus, after analyzing the studies in full, they were cataloged using the software *Microsoft Excel*®, with its data extracted into charts prepared by the authors themselves. The variables collected were title, authors, year of publication, geographic location of the study, methodological design, details of the population, context and phenomenon of interest, including the interdisciplinary/interprofessional dynamics when teams and/or services work in addressing violence prevention.

Data were analyzed qualitatively, more precisely regarding their relevance and pertinence for the topic under study, highlighting methodological aspects, limitations, strengths, comparisons with other studies in the literature and recommendations for future research, according to the narrative synthesis of the main findings. Furthermore, as a way to assess the confidence of the synthesized qualitative findings, the authors followed the recommendations of the ConQual approach. In ConQual, each qualitative study receives a rating of “high” on a scale ranging from high, moderate, low, and very low. From this starting point, each article is rated for its reliability and then for its credibility. Using this tool, the findings of individual studies can be downgraded or maintained in the initial classification^(14)^.

In this regard, reliability is established through the application of the first five questions of the critical evaluation instrument for qualitative studies, related to the adequacy of the research conduct with its objectives and purposes: (1) Is there congruence between the research methodology and the research question or objectives? (2) Is there congruence between the research methodology and the methods used to collect data? (3) Is there congruence between the research methodology and the data representation and analysis? (4) Is there a statement locating the researcher culturally or theoretically? (5) Is the influence of the researcher on the research, and vice versa, addressed?^(13,14)^ According to the number of “yes” answers to the questions, the classification per article is defined: from 4 to 5 “yes” answers – classification remains unchanged; from 2 to 3 “yes” answers – classification lowered by one level; from 0 to 1 “yes” answers – classification lowered by two levels^(13)^.

Credibility, in turn, is scored through the evaluation of the combination of findings included in the associated categories. Thus, credibility is divided into: Unequivocal (U) – presence of findings that are, in fact, directly reported/observed; Credible (C) – plausible evidence in light of the data and theoretical framework; however, as these are interpretative findings, they can be challenged; or Unsupported (US) – presence of findings that are not supported by the data or when unequivocal and credible classifications do not apply. The classification process is defined for each synthesized finding as follows: if all findings unequivocal – classification remains unchanged; if combination of unequivocal/credible findings – classification downgraded by one level; and if findings credible/unsupported: classification downgraded by three levels^(13,14)^.

### Ethical Aspects

As this was a study that used bibliographical information, approval by the Human Research Ethics Committee was not necessary.

## RESULTS

The result of the initial search in the databases resulted in a total of 3,970 studies, of which 776 were found in *PubMed*/*MEDLINE*, 546 in *Embase*, 613 in *CINAHL*, 751 in *PsycInfo*, 536 in *Scopus*, 509 in *Web of Science*, 34 in *LILACS*, seven in *BDENF*, four in the *IndexPsi*, 94 in *SciELO,* and the first hundred in *Google Scholar*. After removing duplicates and applying the inclusion and exclusion criteria, 1,885 studies moved on to the title and abstract reading stage. After this stage, 35 were pre-selected for full reading. Of these, ten were excluded and other three were included after reading of the references, making up the final sample of 28 articles for this review. In Chart 2, the selected articles are presented in ascending order by year of publication, in addition to the description of the country of origin, title, and methodological design.

**Chart 2:** Characteristics of the studies included in the scoping review – Florianópolis, SC, Brazil, 2025.

Country/year	Title	Methodological design
Canada 2000^(15)^	Change among batterers examining men's success stories	Longitudinal (retrospective), descriptive, and exploratory research
USA 2002^(16)^	Group therapy intervention for male batterers: a microethnographic study	Ethnographic, observational research
Canada 2003^(17)^	The men's peace project: a working model with male violence	Longitudinal (retrospective), descriptive, case report type research
Israel 2003^(18)^	Beit Noam: residential program for violent men	Longitudinal (retrospective), descriptive, case report type research
Canada 2004^(19)^	A group for men who abuse their partners: participant perceptions of what was helpful	Descriptive-exploratory study
USA 2006^(20)^	How batterer intervention programs work: participant and facilitator accounts of processes of change	Longitudinal (retrospective), descriptive, and exploratory research
USA 2008^(21)^	Deconstructing male violence against women the men stopping violence community-accountability model	Longitudinal (retrospective), descriptive, case report type research
Israel 2009^(22)^	Control of the self: partner-violent men's experience of therapy	Phenomenological, longitudinal (retrospective), descriptive, and exploratory research
USA 2010^(23)^	Culturally specific treatment for partner-abusive Latino men: a qualitative study to identify and implement program components	Longitudinal (retrospective), descriptive, and exploratory research
United Kingdom 2010^(24)^	A qualitative review of perception of change for male perpetrators of domestic abuse following abuser schema therapy (AST)	Longitudinal (retrospective), descriptive, and exploratory research
Canada 2012^(25)^	An ecological examination of factors influencing men's engagement in intimate partner violence groups	Longitudinal (retrospective), descriptive, case report type research
USA 2013^(26)^	“En el globo tomas conciencia (in group you become aware)”: Latino immigrants' satisfaction with a culturally informed intervention for men who batter	Longitudinal (retrospective), descriptive, and exploratory research
South Africa 2014^(27)^	Intimate partner violence among rural South African men: Alcohol use, sexual decision-making, and partner communication	Longitudinal (retrospective), descriptive, and exploratory research
Australia 2015^(28)^	'I miss my little one a lot': how father love motivates change in men who have used violence	Descriptive-exploratory study
USA 2015^(29)^	A different kind of fraternity: psychological change and group dynamics of male batterers	Longitudinal (retrospective), descriptive-exploratory, comparative research using case studies
Israel 2015^(30)^	Non-violent empowerment: self-help group for male batterers on recovery	Phenomenological, longitudinal (retrospective), descriptive, and exploratory research
Brazil 2016^(31)^	Repercussions of imprisonment for marital violence: discourses of men	Longitudinal (retrospective), descriptive, and exploratory research
United Kingdom 2016^(32)^	“Time out”: a strategy for reducing men's violence against women in relationships?	Longitudinal (retrospective), descriptive, and exploratory research
Brazil 2017^(33)^	La masculinité au travail au sein d’un groupe réflexif pour hommes auteurs de violence contre des femmes	Longitudinal (retrospective), descriptive, case report type research
USA 2017^(34)^	Exploring factors that contribute to positive change in a diverse, group-based male batterer intervention program: using qualitative data to inform implementation and adaptation efforts	Longitudinal (retrospective), descriptive, and exploratory research
Brazil 2018^(35)^	The experience of preventive detention for domestic violence: the discourse of men	Descriptive-exploratory study
Brazil 2019^(36)^	Identificando políticas públicas: Defensoria Pública e homens infratores da Lei Maria da Penha	Exploratory, observational research
USA 2019^(37)^	Male intimate partner violence perpetrators' perceptions of the positives and negatives of peer interactions in group batterer intervention programs	Etnographic, longitudinal (retrospective), descriptive, and exploratory research
USA 2019^(38)^	Male IPV perpetrators' perspectives on facilitation of batterer intervention program: results from a 2-year study	Etnographic, longitudinal (retrospective), descriptive, and exploratory research
Brazil 2020^(39)^	Produção de sentidos em um grupo reflexivo para homens autores de violência	Observational, descriptive, cross-sectional research
Brazil 2020^(40)^	Social technology to prevent intimate partner violence: the vid@ group in actions with men	Descriptive research, experience report type
USA 2020^(41)^	The men's group at St. Pius V: a case study of a parish-based voluntary partner abuse intervention program	Longitudinal (retrospective), descriptive, case report type research
South Africa 2021^(42)^	Steppingstones and creating futures: a group-based approach to addressing violence against women through working with men	Longitudinal (retrospective), descriptive, case report type research

### Characteristics of the Studies Included

The research strategy did not involve a date cutoff, obtaining studies from the year 2000 to 2021, that is, a relatively recent period. Most of the selected studies were published in 2015^(28,29,30)^, 2019^(36,37,38)^ and 2020^(39,40,41)^. Of the 28 manuscripts, ten were developed in the United States of America (USA)^(16,20,21,23,26,29,34,37,38,41)^, six in Brazil^(31,33,35,36,39,40)^, four in Canada^(15,17,19,25)^, three in Israel^(18,22,30)^, two in the UK^(24,32)^, two in South Africa^(27,42)^, and one in Australia^(28)^.

Most studies presented, as a strategy for preventing VAW, secondary and tertiary level approaches applied to men who commit violence. Only two studies^(21,42)^ expanded prevention to the primary level, aiming to prevent incidence, including male volunteers, not necessarily perpetrators of violence and/or judicially referred. Two studies^(31,35)^ presented an exclusively tertiary prevention approach, represented by preventive detention. Chart 3 presents the selected studies with the respective level of prevention highlighted in the identified approaches.

**Chart 3:** Presentation of studies according to approaches at the primary, secondary and/or tertiary prevention levels – Florianópolis, SC, Brazil, 2025.

Prevention-level approach	Studies
Primary Prevention	(21), (42)
Secondary Prevention	(15), (16), (17), (18), (19), (20), (21), (22), (23), (24), (25), (26), (27), (28), (29), (30), (32), (33), (34), (36), (37), (38), (39), (40), (41), (42)
Tertiary Prevention	(15), (16), (17), (18), (19), (20), (21), (22), (23), (24), (25), (26), (27), (28), (29), (30), (32), (33), (34), (36), (37), (38), (39), (40), (41), (42)

### Primary, Secondary, and Tertiary Prevention Approaches Applied to Men who Commit Violence Against Women

The approaches were mostly carried out in groups^(15#x2013;23,25,26,27,28,29,30,33,34,36,37,38,39,40,41,42)^. An intervention occurred exclusively in the form of individual therapy^(24)^, and three others mixed the two previous approaches^(18,22,28)^. Two studies described the pretrial detention intervention^(31,35)^ and one, the teaching of a technique to avoid marital conflicts^(32)^. In group approaches, the number of sessions varied, with a minimum of eight weekly sessions^(17)^ and a maximum of 52 weekly sessions^(23,34)^. Actions with 16 sessions^(18,33,37,38)^ and 24 weekly sessions^(19,37,38)^ were prevalent. The meetings lasted two hours each in most of the studies described^(16,17,19,26,29,37,38,39,41)^. The interventions used references such as feminist^(15,25,26)^ and gender^(22,23,27,39,42)^ theories, cognitive-behavioral therapy^(20,22,24,25,26)^, Paulo Freire’s approach^(26,33,40,42)^, and Duluth model^(20,29)^.

Of the studies describing men’s adherence patterns, seven^(15,22,29,33,34,37,38)^ were both through judicial referral and voluntarily, three^(17,36,39)^ only judicially, and three others^(19,21,41)^ open to the public on a voluntary basis. As for the number of men involved in the studies and interventions, the minimum was three^(16)^ and the maximum 177^(32)^, the latter also being carried out with the men’s partners. Among the themes addressed in the interventions, the following stood out: parenting^(18,20,21,23,25,28,34,39,41)^, impacts of violence on children and families^(18,22,23,28,41)^, communication and dialogue in conflict resolution^(16,21,22,39,41)^, anger management^(16,22,39)^, conception and contraception^(27,28,42)^, expectations^(28,39,42)^ and gender equality^(23,27)^, empathy^(16,28)^, and abuse of alcohol and other drugs^(23,39)^. In some cases, referrals were made to other services, such as Alcoholics Anonymous^(19,35)^ and mental health devices^(34)^.

### Interdisciplinary/Interprofessional Dynamics When Teams and/or Services Work to Address Violence Prevention

Few studies^(19,25,38,39)^ described the interdisciplinary/interprofessional dynamics when teams and/or services work in the VAW prevention approach. Of these, three were composed of mixed groups, with female and male facilitators^(19,25,38)^, one for men only^(33)^ and one for women only^(39)^. The training of facilitators involved areas such as psychology, social assistance, and other fields of social sciences^(25,33,39)^. A study sought to understand men’s perceptions of the facilitators of the group intervention program in which they participated, so that, initially challenging, facilitation by women was important to avoid making the group unilateral, in addition to favoring the expression of male feelings and the witnessing of positive interactions between men and women, therefore recognizing the importance of mixed groups. Furthermore, the non-judgment on the part of the facilitators helped in mobilizing the change process^(38)^.

### Repercussions of Approaches to Men Who Commit Violence and Their Families

In general, the men participating in the studies evaluated approaches to violence prevention positively^(19,20,23,24,28,36,39)^, highlighting repercussions such as: responsibility for one’s behavior^(15,18,19,21,22 23,32,34,37,41)^, mobilization for the process of change^(16,20,22,23,25,26,37,40,41)^, communication improvement^(15,19,22,24,27,32)^, and in the relationship with children, partners and family^(23,26,27,28,39)^, anger management^(22,24,42)^, interpersonal learning through stories shared by other men^(30,37)^, development of empathy^(15,19)^, anxiety relief by expressing emotions in a safe environment^(23,24)^, reduction of alcohol and other substance intake^(27,42)^, control of abusive behaviors^(19,26)^, decrease in intimate partner violence^(42)^ *,* and interruption of physical violence^(32)^. One of the themes most mentioned in the actions and which generated the greatest commotion was the one regarding parenthood^(18,20,21,23,25,28,34,39,41)^. The theme of parenting was associated both with the desire to provide a healthier family environment for children and not to repeat the same parental model received by the father of the man who committed violence^(25,28,34)^.

Two studies found that there was no reflection on the part of the male participants^(31,35)^. It is noteworthy that both were dealing with preventive detention, in which anger and feelings of injustice prevailed among them. Men were also reported to feel social stigma when they belonged to a violent population^(22,31)^ and psycho-emotional symptoms, such as headache, hypertension, phobia and depression^(31)^. One study reported participants’ intention to use prevention action as a way to favor their trial^(33)^. Furthermore, cases of recidivism were described, one in the study developed in 2003, in Canada, in which a case of violence was reported among the seven former participants of the group^(17)^, and another, carried out in 2016, in the USA, in which one of the 48 men participating caused an episode of violence while participating in the group^(29)^.

Some studies also conducted interviews with the men’s wives and ex-wives, as well as some family members^(17-19,21,25,32,36)^, highlighting the dissatisfaction of some women with prevention approaches^(18,32,36)^. Specifically, teaching the technique of “taking a time out”, that is, withdrawing from a conflict situation for a period of time, showed the potential to improve communication, but it was also used inappropriately, as a new strategy for men to control women. In some situations, women had to insist that the man take a break, so they were forced to leave, or the men left the scene, but did not reflect on what had happened^(32)^.

The two studies^(21,42)^ that expanded their prevention strategies to the primary level demonstrated emotional relief through the recognition and sharing of a broad male socialization process that often limits the spaces for welcoming men’s feelings, undermining the possibilities for reflection. Thus, these strategies had the ability to reduce shame and provide nonviolent techniques for dealing with anger^(42)^. Moreover, the inclusion of these men’s peers, friends and family members was identified as an important intervention for accountability, encouraging mutual and systemic change, such as active participation as agents of community change^(21,42)^.

Finally, Chart 4 shows the summary of the findings according to the ConQual Score. This analysis resulted in seven summaries, in which the ConQual Score varied between high, moderate, and low. The classification provided a moderate degree of confidence for the synthesized finding due to reliability issues (lack of clarification about the methods used in data collection and analysis). In four summaries, credibility was assessed as moderate, since the findings were classified as unequivocal and credible, that is, not all of them significantly represented the findings presented in the primary studies.

**Chart 4:** Summary of findings and ConQual Score – Florianópolis, SC, Brazil, 2025.

Title: Prevention approaches aimed at men who commit violence against women			
Population: men who commit violence against women Concept: primary, secondary, and tertiary prevention approaches Context: violence against women			
**Summary of findings**	**Reliability**	**Credibility**	**ConQual Score**
Positive repercussions of approaches to men, such as changes in their behavior, communication and relationships^(15,16,18,19,20,21,22,23,24,25,26,27,28,30,32,33,34,36,37,39,40,41,42)^	High	Total: 23 (10I + 13C)	Moderate
Lack of reflection on the part of men, especially in contexts of pre-trial detention^(31,35)^	High	Total: 2 (2I)	High
Reports of repeated violent acts during the men’s participation in the approaches^(17,29)^	Moderate	Total: 2 (2C)	Low
Dissatisfaction with prevention approaches by men’s partners^(18,32,36)^	High	Total: 3 (3I)	High
Approaches based on feminist and gender theories(15,22,23,25,26,27,39,42)	High	Total: 8 (4I + 4C)	Moderate
Importance of mixed teams and non-judgment by facilitators of group approaches^(19,25,38)^	High	Total: 3 (1I + 2C)	Moderate
Primary prevention approaches have shown potential for emotional relief, accountability, and community transformation, especially when including men’s peers, friends, and family members^(21,42)^	High	Total: 2 (1I + 1C)	Moderate

## DISCUSSION

Primary prevention actions aim to modify social beliefs and involve actions such as anti-violence campaigns and empowerment programs, reducing risk factors and incorporating health promotion strategies and universal education for the general public. The secondary level, in turn, implemented when a primary intervention fails or does not occur, seeks to prevent the recurrence of violence through screening programs or referrals to judicial services, for example. Finally, tertiary prevention corresponds to adjustment processes to alleviate problems resulting from violence through support for physical and mental health, in addition to social and legal care^(1,43)^. The process of changing deeply ingrained social norms is slow and requires interventions at the secondary and tertiary levels. However, given the high global burden of VAW and the saturation of certain secondary and tertiary resources, such as for mental health, legal and housing, scaling up of primary prevention is crucial for reducing VAW in the long term^(1,44)^.

In recent years, several countries have invested in the involvement of men and boys as a primary prevention strategy for VAW^(45)^. It is believed that efforts to prevent VAW should address men, given that masculinity plays a critical role in the formation of violence, so that, to a large extent, men are the perpetrators and, therefore, have the potential to play an active role in stopping and preventing violence^(6,46)^. Therefore, addressing hegemonic masculine norms is an important part of preventing gender-based violence, considering that such masculinity is not innate, but constructed and, therefore, is subject to change^(47)^.

Hegemonic masculinity is less likely to support gender equity and more likely to be involved in perpetrating VAW, while being aware of male privilege and supporting gender equity is associated with greater willingness to engage in violence-preventing behaviors^(47)^. Thus, involving men in violence prevention can have a positive impact not only on the lives of women and girls, but on the lives of men and boys, freeing them from harmful and rigid aspects of traditional masculinities^(6,46)^. However, often the fear of being labeled as a perpetrator and hegemonic masculine norms themselves prevent men from discussing their feelings. Furthermore, the modification and interruption of benefits conferred by unequal power and gender relations generates resistance to change on the part of men^(46)^.

The theme of parenting, as one of the most frequently mentioned in the approaches described in this review, generated commotion, reflection, and motivation for change. Given that suffering violence is often cited as a factor that contributes to the likelihood of perpetrating violence, it is not uncommon for men who perpetrate violence to be children who witnessed and/or experienced violent family situations^(46,48)^. Children who witness situations of violence experience psychosocial, emotional and cognitive effects, in terms of memory and learning, and may reproduce negative behaviors. This understanding by the men participating in the interventions can generate authentic motivation when they perceive the damage and breakdown in the relationship with their children and partners. Such internal motivation has greater potential for adherence to change when compared to so-called extrinsic motivations, such as those imposed by law and court orders, which often do not generate the same reflection^(49)^.

This review highlighted tertiary prevention approaches, represented by preventive detention. However, it was found that these interventions did not generate reflection, on the contrary. Anger and feelings of injustice were also present in a study carried out with men who experienced preventive detention^(50)^. Regarding recidivism, a study carried out in a reflective group for male perpetrators of violence in Brazil found that 19.7% were repeat offenders before participating in the intervention. After participating, only 1.3% of participants were prosecuted again for the same act^(51)^. However, it is understood that more studies on the evaluation of these actions are necessary, given the little scientific production in this area^(52)^.

In turn, actions with a gender-transformative approach have achieved positive results in reducing intimate partner violence, with a reduction in victimization and perpetration at individual, family, and community levels of physical, sexual, and/or psychological violence, especially when based on the sociocultural context of the community^(53)^. Therefore, interventions that encourage men to reflect and recognize how gender shapes norms, discourse, and subjectivity in their lives, critically reflecting on hegemonic masculinity, are essential^(54)^.

In addition to work focused on reflection and gender-equitable attitudes, the Duluth model, based on a psychoeducational model and feminist theories, highlights the importance of focusing on respect, equality, and accountability, so as to modify patriarchal and unequal cultural norms in society^(55)^. Unlike interventions focused on re-education, recovery, and/or resocialization, which fit into a vertical perspective of learning, working with men who are perpetrators of violence in group, reflective processes oriented towards accountability has as its purpose the self-understanding and self-criticism of their personal history, enabling, through the group and dialogical space, collective constructions for the re-elaboration of the current model of masculinity^(56)^. The establishment of mixed facilitation, that is, with men and women, can also encourage reflection and gender-equitable attitudes on the part of men who commit violence, by allowing the listening and perspective of the facilitating woman who, in a certain way, represents his (ex)partner^(46,57)^.

Although work with men in violence prevention is growing, actions addressing the male public beyond prevention objects, but also as agents of prevention and social change, are still scarce. It is understood that interventions for male perpetrators of VAW are fundamental; however, they need to be articulated with primary level actions to promote healthy gender norms, engaging boys from an early age to men in general, including authorities, such as police officers and religious leaders^(45,46)^. Furthermore, it is suggested that work be expanded to include women and other family members, considering the relational nature of gender-based violence^(49,51)^.

Supporting and empowering professionals in education, health and social services to question their own biases about gender stereotypes and norms; implementing sexuality education in schools, universities, and community programs; inquiring about intimate relationships and providing education about safe and healthy relationships; empowering men and women to recognize and prevent violence in relationships; addressing violence in smoking cessation and alcohol use reduction groups; and promoting positive, nonviolent, and respectful forms of parenting in parents groups during prenatal and/or postnatal care are some examples of primary VAW prevention interventions and health promotion strategies^(1,47,49)^.

Despite the potential to involve men in violence prevention, there are a number of challenges to be overcome, such as human resources, theoretical foundations, and political will^(46)^. Health professionals have reported difficulty and lack of preparation to deal with these situations, especially in cases of psychological violence, where the signs are not as easily identifiable as in physical violence, for example^(5)^. Therefore, it is essential to incorporate the intersectional and gender perspective into professional training to address and overcome violence, as well as multi and interdisciplinary action^(58)^.

As a limitation, we can mention the lack of description of the interdisciplinary/interprofessional dynamics when teams and/or services work in the approach to violence prevention, which highlights the need for future research in this regard. It is expected that the information and reflections presented in this scoping review will be taken into consideration by researchers, especially during the methodological construction of subsequent studies, as well as by professionals involved in health practices and public policy makers, to strengthen and propose recommendations for better intervention in the prevention of violence and the promotion of violence-free marital and family relationships.

## CONCLUSION

This review allowed the mapping of primary, secondary, and tertiary prevention approaches applied to men who commit violence against women. Thus, it was found that most studies presented secondary and tertiary level approaches applied to men who commit violence, with only two studies qualifying for the primary level of prevention.

The interventions, in general, followed the group approach, with the potential to be replicated in other services, including health services and for other audiences, as long as they have the theoretical and human support for this, expanding the actions to the primary level of prevention, which is still in its infancy. Only five studies described interdisciplinary dynamics, so that the use of groups facilitated by men and women was identified, mostly from the areas of psychology, social assistance, and other fields of social sciences.

In the Brazilian reality, group interventions, widely used, as seen in this review, gain prominence in Primary Health Care, which has the potential to include men and women and address, in an interdisciplinary way, sensitive issues, such as violence, parenting, and alcohol abuse, for example, favoring violence prevention actions, especially those at the primary level.

## Data Availability

The dataset supporting the findings of this study is not publicly available, however, the full dataset supporting the findings of this study is available upon request to the corresponding author.
