# Supplementary material for: Prevention approaches aimed at men who commit violence against women: a scoping review
Source: Rev Esc Enferm USP. 2025 Jul 7;59:e20250036. doi: 10.1590/1980-220X-REEUSP-2025-0036en (PMC12245394; doi:10.1590/1980-220X-REEUSP-2025-0036en)
Supplement: Translation

# Translation: Abordagens de prevenção voltadas aos homens autores de violência contra mulheres: revisão de escopo

**DOI:** 10.1590/1980-220X-REEUSP-2025-0036pt

**Authors:** Victoria Leslyê Rocha Gutmann, Gisele Cristina Manfrini, Tereza Maria Mendes Diniz de Andrade Barroso, Camila Daiane Silva, Sheila Rubia Lindner, Sandra Mara Corrêa

**Affiliations:** 1 Universidade Federal de Santa Catarina, Programa de Pós-Graduação em Enfermagem, Florianópolis, SC, Brasil.; 2 Universidade Federal de Santa Catarina, Departamento de Enfermagem, Florianópolis, SC, Brasil.; 3 Escola Superior de Enfermagem de Coimbra, Coimbra, Portugal.; 4 Universidade Federal do Rio Grande, Escola de Enfermagem, Rio Grande, RS, Brasil.; 5 Universidade Federal de Santa Catarina, Departamento de Saúde Pública, Florianópolis, SC, Brasil.

**Keywords:** Homens, Prevenção Primária, Prevenção Secundária, Prevenção Terciária, Violência contra a Mulher

## Abstract

**Objetivo:**

Mapear as abordagens de prevenção primária, secundária e terciária aplicadas aos homens autores de violência contra mulheres.

**Método:**

Revisão de escopo qualitativa, conforme diretrizes do JBI. A busca foi realizada de julho a outubro de 2022 nas bases MEDLINE, Embase, CINAHL, PsycInfo, Scopus, Web of Science, LILACS, BDENF, IndexPsi, SciELO e Google Scholar, sem restrição quanto a área geográfica, data de publicação e/ou idioma. Todos os estudos foram avaliados quanto à confiabilidade e credibilidade, segundo o Score ConQual.

**Resultados:**

Foram selecionados 28 estudos, a maioria com abordagem de nível secundário e terciário. Apenas dois estudos enquadraram-se na prevenção primária. As intervenções, majoritariamente, foram realizadas em grupo. A dinâmica interdisciplinar/interprofissional, raramente descrita, envolveu áreas como psicologia, assistência social e demais campos das ciências sociais.

**Conclusão:**

As intervenções, de modo geral, seguiram a abordagem grupal, com potencialidade para serem replicadas em outros serviços, inclusive de saúde e para outros públicos, desde que possuam aporte teórico e humano para tal, ampliando as ações para o nível primário de prevenção.

## INTRODUÇÃO

A Violência contra as Mulheres (VCM) envolve uma evolução complexa que transcende diferentes fases da vida, tornando-se uma problemática social e global, visibilizada diariamente em dados significativos de mulheres que sofrem e são mortas por serem mulheres^(1)^. Globalmente, cerca de 30% das meninas e mulheres, entre 15 e 49 anos, afirmaram terem vivenciado, pelo menos uma vez, violência física e/ou sexual por parceiro íntimo, com maior prevalência nos países de baixa renda. No Brasil, essa taxa é de 23%^(2)^. Apenas em 2022, o país registrou 51.407 casos de violência física. O local mais comum na ocorrência da VCM foi a residência, em mais de 80% dos casos, de forma que os homens foram os principais autores, em 86,6% dos registros. Quanto aos casos de feminicídios, estima-se uma taxa de 3,5 casos para cada 100 mil mulheres, em que, novamente, os homens, em específico namorados e cônjuges, foram os autores principais^(3)^.

Os danos da violência, quando não fatais, vão desde os físicos, como lesões e ferimentos, até os psicológicos, envolvendo comprometimento psicoemocional das mulheres, como baixa autoestima e transtornos mentais comuns, como depressão, ansiedade, distúrbios do sono, estresse pós-traumático e ideação suicida, além de hiperatividade, dificuldade de atenção e aprendizagem, padrão alimentar inadequado e uso e/ou abuso de drogas e medicamentos^(4)^. Tais repercussões também incidem sobre a família e as pessoas próximas das mulheres em situação de violência, além da sociedade como um todo, tornando-se uma demanda cada vez mais presente nos serviços de saúde. Desse modo, a VCM constitui-se uma preocupação de nível global e um importante problema de saúde pública, necessitando, portanto, de ações que visem o seu enfrentamento e, especialmente, sua prevenção^(5)^.

Nesse sentido, as abordagens de prevenção são divididas nos níveis primário, secundário e terciário. A prevenção primária compreende as ações realizadas antes da ocorrência da violência, de modo a prevenir a incidência e a prevalência dos casos, isto é, a perpetração inicial ou vitimização. A prevenção secundária, por sua vez, busca reduzir a violência que já está em andamento, identificando indivíduos e famílias em risco por meio da existência de crises situacionais desencadeadoras de violência e, assim, centra-se em uma resposta imediata. Por último, a prevenção terciária baseia-se em respostas em longo prazo, visando ofertar a assistência após o ocorrido, evitando a reincidência^(6)^. Embora necessárias, as respostas secundárias e terciárias, por si só, não são capazes de reduzir a incidência da VCM, tampouco prevenir ou intervir totalmente. Abordagens primárias fornecem uma perspectiva ampla e holística na atuação diante de problemas relacionais e familiares^(1)^.

Assim, tem-se buscado ir além dos esforços de prevenção secundária e terciária para permitir maiores oportunidades de prevenção primária. Acredita-se que uma estrutura de relacionamento saudável oferece amplas oportunidades para a prevenção da violência, seja promovendo o vínculo entre a criança e o cuidador, incentivando relacionamentos de orientação entre jovens e adultos ou ofertando recursos financeiros com vistas a diminuir o estresse econômico familiar^(7)^. Esse último tópico, especialmente, tem sido associado com a permanência de mulheres em situações de violência devido à dependência financeira. Tal dependência ocorre quando o companheiro é o provedor ou exerce o controle da renda familiar. Ainda, cenários de emprego e salário precários também contribuem para o aumento da vulnerabilidade das mulheres^(5)^.

Além das variáveis individuais, psicológicas e relacionais, os homens, especificamente, são fruto de uma construção sociocultural de gênero que normatiza e naturaliza o poder masculino sobre o feminino, alicerçado em preceitos patriarcais que, muitas vezes, os aproximam de atos ditos viris e, consequentemente, violentos. Esse fato se expressa em situações em que o homem, ao perceber sua identidade masculina ameaçada, pode vir a lançar mão da violência^(8)^. Logo, a prevenção da violência também deve considerar a transformação e desconstrução de formas cristalizadas de pensar, agir e sentir da masculinidade hegemônica, bem como de sua função social e afetiva na relação com as mulheres e filhos^(9)^. Ou seja, a superação da violência requer a construção de formas alternativas de ser homem, ao encontro de relações conjugais harmoniosas, equitativas e não violentas^(10)^. Todavia, ainda são escassos os estudos que envolvem os homens autores de violência, especialmente com caráter preventivo e/ou voltados à mensuração da efetividade de abordagens que envolvam esse grupo^(11)^.

A Organização Mundial da Saúde reconhece a VCM como uma grave violação dos direitos humanos e um obstáculo para atingir os Objetivos do Desenvolvimento Sustentável (ODS) e, por isso, tem incentivado o trabalho e estudos junto aos homens para a promoção da equidade e igualdade de gênero^(10)^. Quanto aos ODS, destacam-se o 5 e o 16, que dizem respeito, respectivamente, à igualdade de gênero para combater as desigualdades, incluindo a violência contra as mulheres, e a promoção de sociedades pacíficas. Especificamente o ODS 5, que trata do alcance da igualdade de gênero e do empoderamento feminino, caracteriza-se como um compromisso histórico no enfrentamento, na prevenção e na eliminação de todas as formas de violência baseadas no gênero, tendo em vista a persistência e os efeitos adversos dessa problemática^(12)^.

Assim, para verificar o corpo de evidências acerca das abordagens para prevenção da VCM, surgiu a necessidade de realizar uma revisão da literatura, especificamente uma revisão de escopo, de modo a fornecer uma visão geral acerca das ações e estratégias que visam a prevenção primária, secundária e terciária da VCM, especialmente voltadas aos homens autores de violência. Dessa forma, objetivou-se: mapear as abordagens de prevenção primária, secundária e terciária aplicadas aos homens autores de violência contra mulheres.

## MÉTODO

### Desenho do Estudo

Trata-se de uma revisão de escopo, realizada conforme as diretrizes metodológicas do *Joana Briggs Institute* (JBI), seguindo as etapas: identificação da questão e objetivo da pesquisa; estratégia de busca; seleção dos estudos, de acordo com os critérios pré-estabelecidos; mapeamento e extração dos dados; análise da evidência; e apresentação sumarizada dos resultados. Esta metodologia busca fornecer uma visão geral ou um mapa das evidências e possíveis lacunas do conhecimento, bem como explorar a extensão da literatura sobre determinado tópico, abrindo caminho para pesquisas futuras com direcionamento mais específico^(13)^.

Também foram consideradas as recomendações do *Preferred Reporting Items for Systematic Reviews and Meta-Analyses* (PRISMA-ScR) que inclui o uso de um *checklist* para elaboração e relato dos passos metodológicos do protocolo desta revisão^(13)^. A revisão foi cadastrada na plataforma *Open Science Framework*, com identificação DOI: http://dx.doi.org/10.17605/OSF.IO/XGVA5.

### Critérios de Seleção

Para a definição da questão do estudo, adotou-se o acrônimo PCC, sendo P - População = homens autores de violência contra mulheres; C - Conceito = abordagens de prevenção primária, secundária e terciária; e C - Contexto = violência contra mulheres. Desse modo, formulou-se a seguinte questão: “Quais abordagens de prevenção primária, secundária e terciária são aplicadas aos homens autores de violência contra mulheres?”.

A partir disso, foram definidos os seguintes critérios de inclusão: artigos originais de pesquisa, do tipo qualitativa, que tratavam das abordagens de prevenção primária, secundária e/ou terciária voltadas a homens autores de violência, de qualquer nacionalidade, raça ou cor. Não foi estabelecido limite de área geográfica, data de publicação e/ou idioma, exceto se versões exclusivamente de texto completo em mandarim. Os critérios de exclusão aplicados foram teses, dissertações e artigos duplicados, incompletos e/ou que não correspondiam ao tema da pesquisa.

A busca foi realizada em 11 bancos de dados indexados e na literatura cinzenta, sendo: *PubMed*/*MEDLINE*, *Embase*, *CINAHL*, *PsycInfo*, *Scopus*, *Web of Science*, *LILACS*, *BDENF*, *IndexPsi*, *SciELO* e *Google Scholar*. Como complementação da estratégia de busca, foi realizada a consulta das referências textuais dos estudos selecionados para leitura integral, que puderam ser incluídos caso atendessem aos critérios de inclusão e a questão e objetivo do estudo.

Destaca-se que a sintaxe de busca foi adaptada para cada banco de dados, em conformidade com seu formato. As palavras contidas nos títulos e resumos de artigos relevantes e os termos de indexação utilizados para descrever os artigos foram analisados por duas autoras e uma bibliotecária, de modo a compor a estratégia, em conformidade com cada item PCC, selecionando um conjunto de descritores disponíveis nos Descritores de Ciências da Saúde (DeCS), no *Medical Subject Headings* (MeSH), além de palavras-chave, em português, inglês e espanhol. A estratégia de busca foi elaborada por meio da combinação dos termos do DeCS, *MeSH* e palavras-chave, com a aplicação dos operadores booleanos E/*AND* e OU/*OR*. As estratégias de busca e a associação dos descritores com os operadores booleanos, para cada base de dados, podem ser consultadas no Quadro 1.

**Quadro 1:** Estratégia de busca nas bases de dados – Florianópolis, SC, Brasil, 2025.

Bases de dados	Estratégia de busca
*Pubmed/MEDLINE*	((“Violence Against Women“ OR “Crimes against Women“ OR “Gender-Based Violence“[Mesh] OR “Gender-Based Violence“ OR “Domestic Violence“[Mesh] OR “Domestic Violence“ OR “Intimate Partner Violence“[Mesh] OR “Intimate Partner Violence“ OR “Dating Violence“ OR “Intimate Partner Abuse“) AND (“Primary Prevention“[Mesh] OR “Primary Prevention“ OR “Prevention“ OR “Preventive Health Services“[Mesh] OR “Preventive Health Services“ OR Preventive* OR “Secondary Prevention“[Mesh] OR “Secondary Prevention“ OR “Tertiary Prevention“[Mesh] OR “Tertiary Prevention“) AND (“Men“[Mesh] OR “Men“[Title] OR “Man“[Title] OR “Male“[Title]))
*Embase*	((“Violence Against Women“ OR “Crimes against Women“ OR “Gender-Based Violence“ OR “Domestic Violence“ OR “Intimate Partner Violence“ OR “Dating Violence“ OR “Intimate Partner Abuse“) AND (“Primary Prevention“ OR “Prevention“ OR “Preventive Health Services“ OR Preventive* OR “Secondary Prevention“ OR “Tertiary Prevention“) AND (“Men“/de OR “Men“:ti OR “Man“:ti OR “Male“:ti))
*CINAHL* *PsycInfo* *Scopus*	((“Violence Against Women“ OR “Crimes against Women“ OR “Gender-Based Violence“ OR “Domestic Violence“ OR “Intimate Partner Violence“ OR “Dating Violence“ OR “Intimate Partner Abuse“) AND (“Primary Prevention“ OR “Prevention“ OR “Preventive Health Services“ OR Preventive* OR “Secondary Prevention“ OR “Tertiary Prevention“) AND (“Men“ OR “Men“ OR “Man“ OR “Male“))
*Web of Science*	NOFT((“Violence Against Women“ OR “Crimes against Women“ OR “Gender-Based Violence“ OR “Domestic Violence“ OR “Intimate Partner Violence“ OR “Dating Violence“ OR “Intimate Partner Abuse“) AND (“Primary Prevention“ OR “Prevention“ OR “Preventive Health Services“ OR Preventive* OR “Secondary Prevention“ OR “Tertiary Prevention“) AND (“Men“ OR “Men“ OR “Man“ OR “Male“))
*LILACS* *BDENF* *IndexPsi* *SciELO*	((“Violência contra a Mulher“ OR “Crimes contra a Mulher“ OR “Crimes contra as Mulheres“ OR “Violência contra as Mulheres“ OR “Violência de Gênero“ OR “Violência Doméstica“ OR “Violência por Parceiro Íntimo“ OR “Violencia contra la Mujer“ OR “Crímenes contra la Mujer“ OR “Crímenes contra las Mujeres“ OR “Violencia contra las Mujeres“ OR “Violencia de Pareja“ OR “Violence Against Women“ OR “Crimes against Women“ OR “Gender-Based Violence“ OR “Domestic Violence“ OR “Intimate Partner Violence“ OR “Dating Violence“ OR “Intimate Partner Abuse“) AND (“Prevenção Primária“ OR “Prevenção“ OR “Serviços Preventivos de Saúde“ OR Preventiv* OR “Prevención Primaria“ OR “Prevención“ OR “Servicios Preventivos de Salud“ OR “Primary Prevention“ OR “Prevention“ OR “Preventive Health Services“ OR “Prevenção Secundária“ OR “Prevenção Terciária“ OR “Prevención Secundaria“ OR “Prevención Terciaria“ OR “Secondary Prevention“ OR “Tertiary Prevention“) AND (“Homens“ OR “Homem“ OR “masculino“ OR “Hombres“ OR “Hombre“ OR “Men“ OR “Man“ OR “Male“)
*Google Scholar*	((“Violência contra a Mulher“ OR “Violência de Gênero“ OR “Violência Doméstica“ OR “Violence Against Women“ OR “Gender-Based Violence“ OR “Domestic Violence“) AND (Prevenção OR Prevention) AND (“Homens“ OR “Homem“ OR “Men“ OR “Man“))

### Coleta de Dados

A coleta de dados foi realizada entre julho e outubro de 2022, de modo que todos os estudos recuperados nas bases de dados e os cem primeiros da literatura cinzenta foram exportados para o gerenciador de referências *EndNote Web*®, versão gratuita, no qual os resultados duplicados foram excluídos. Após, para verificar a correspondência dos artigos à questão de pesquisa, a leitura dos títulos, resumo e descritores foi realizada na plataforma *Rayyan Web*®.

O método duplo cego foi seguido e a inclusão ou exclusão ocorreu inicialmente pelo título, seguido pelo resumo, e por fim, pela leitura integral do texto. Após a confirmação de inclusão do artigo, os revisores também realizaram a busca de estudos potencialmente úteis nas referências destes. Em caso de divergência, foi prevista a possibilidade de consultar um terceiro autor, o que não foi necessário durante o processo. Os resultados da busca e do processo de identificação e seleção dos estudos, bem como os motivos de exclusão, foram registrados e relatados no fluxograma PRISMA-ScR, conforme a Figura 1.

**Figura 1:** Fluxograma do processo de seleção dos artigos incluídos.

### Análise e Tratamento dos Dados

A partir da leitura completa dos artigos incluídos, os revisores destacaram e sumarizaram as informações que responderam à questão e ao objetivo da revisão. Assim, após a análise dos estudos na íntegra, eles foram catalogados com uso do *software Microsoft Excel*®, tendo seus dados extraídos para quadros elaborados pelos próprios autores. As variáveis coletadas foram título, autores, ano de publicação, localização geográfica do estudo, desenho metodológico, detalhes da população, do contexto e do fenômeno de interesse, incluindo a dinâmica interdisciplinar/interprofissional quando da atuação de equipes e/ou serviços na abordagem da prevenção da violência.

Os dados foram analisados qualitativamente, mais precisamente quanto à relevância e pertinência para a temática em estudo, destacando-se aspectos metodológicos, limitações, fortalezas, comparações com outros estudos na literatura e recomendações para pesquisas futuras, de acordo com a síntese narrativa dos principais achados. Além disso, como forma de avaliar a confiança das descobertas qualitativas sintetizadas, os autores seguiram as recomendações da abordagem ConQual. No ConQual, cada estudo qualitativo recebe uma classificação de “alta”, em uma escala que varia entre alta, moderada, baixa e muito baixa. A partir deste ponto de partida, cada artigo é classificado quanto a sua confiabilidade e, em seguida, sua credibilidade. Por meio desta ferramenta, os achados dos estudos individuais podem ser rebaixados ou mantidos na classificação inicial^(14)^.

Nesse sentido, a confiabilidade é estabelecida por meio da aplicação das cinco primeiras perguntas do instrumento de avaliação crítica para estudos qualitativos, relacionadas à adequação da condução da pesquisa com seus objetivos e propósitos: (1) Há congruência entre a metodologia da pesquisa e a questão ou objetivos da pesquisa? (2) Há congruência entre a metodologia da pesquisa e os métodos usados para coletar dados? (3) Há congruência entre a metodologia da pesquisa e a representação e análise de dados? (4) Há uma declaração localizando o pesquisador culturalmente ou teoricamente? (5) A influência do pesquisador na pesquisa, e vice-versa, é abordada^(13,14)^. Conforme o número de respostas “sim” para as questões, a classificação por artigo é definida: de 4 a 5 respostas “sim” – classificação permanece inalterada; de 2 a 3 respostas “sim” – classificação rebaixada em um nível; de 0 a 1 respostas “sim” – classificação rebaixada em dois níveis^(13)^.

A credibilidade, por sua vez, é pontuada por meio da avaliação da combinação dos achados incluídos nas categorias associadas. Dessa forma, a credibilidade divide-se em: Inequívoca (I) – presença de descobertas que são, de fato, diretamente relatadas/observadas; Credível (C) – evidências plausíveis à luz dos dados e da estrutura teórica, porém, por se tratar de descobertas interpretativas, podem ser contestadas; ou Não Suportada (NS) – presença de descobertas que não são suportadas pelos dados ou quando classificação inequívoca e credível não se aplicam. O processo de classificação é definido para cada descoberta sintetizada, da seguinte maneira: se todos os achados inequívocos – classificação permanece inalterada; se combinação de achados inequívocos/credíveis – classificação rebaixada em um nível; e se descobertas credíveis/não suportadas: classificação rebaixada em três níveis^(13,14)^.

### Aspectos Éticos

Por se tratar de um estudo que utilizou informações bibliográficas, não foi necessária a aprovação pelo Comitê de Ética em Pesquisa com Seres Humanos.

## RESULTADOS

O resultado da busca inicial nas bases de dados resultou em um total de 3.970 estudos, dos quais 776 foram encontrados no *PubMed*/*MEDLINE*, 546 na *Embase*, 613 na *CINAHL*, 751 na *PsycInfo*, 536 na *Scopus*, 509 na *Web of Science*, 34 na *LILACS*, sete na *BDENF*, quatro na *IndexPsi*, 94 na *SciELO* e os cem primeiros no *Google Scholar*. Após a remoção dos duplicados e a aplicação dos critérios de inclusão e exclusão, 1.885 estudos passaram para a etapa da leitura dos títulos e resumos. Após essa etapa, 35 foram pré-selecionados para leitura integral. Desses, dez foram excluídos e outros três foram incluídos a partir da leitura das referências, compondo a amostra final de 28 artigos desta revisão. No Quadro 2, os artigos selecionados são apresentados em ordem crescente por ano de publicação, além da descrição do país de origem, título e desenho metodológico.

**Quadro 2:** Características dos estudos incluídos na revisão de escopo – Florianópolis, SC, Brasil, 2025.

País/ano	Título	Desenho metodológico
Canadá 2000^(15)^	Change among batterers examining men's success stories	Pesquisa longitudinal (retrospectiva), descritiva e exploratória
EUA 2002^(16)^	Group therapy intervention for male batterers: a microethnographic study	Pesquisa etnográfica, observacional
Canadá 2003^(17)^	The men's peace project: a working model with male violence	Pesquisa longitudinal (retrospectiva), descritiva, do tipo relato de caso
Israel 2003^(18)^	Beit Noam: residential program for violent men	Pesquisa longitudinal (retrospectiva), descritiva, do tipo relato de caso
Canadá 2004^(19)^	A group for men who abuse their partners: participant perceptions of what was helpful	Estudo descritivo-exploratório
EUA 2006^(20)^	How batterer intervention programs work: participant and facilitator accounts of processes of change	Pesquisa longitudinal (retrospectiva), descritiva e exploratória
EUA 2008^(21)^	Deconstructing male violence against women the men stopping violence community-accountability model	Pesquisa longitudinal (retrospectiva), descritiva, do tipo relato de caso
Israel 2009^(22)^	Control of the self: partner-violent men’s experience of therapy	Pesquisa do tipo fenomenológica, longitudinal (retrospectiva), descritiva e exploratória
EUA 2010^(23)^	Culturally specific treatment for partner-abusive latino men: a qualitative study to identify and implement program components	Pesquisa longitudinal (retrospectiva), descritiva e exploratória
Reino Unido 2010^(24)^	A qualitative review of perception of change for male perpetrators of domestic abuse following abuser schema therapy (AST)	Pesquisa longitudinal (retrospectiva), descritiva e exploratória
Canadá 2012^(25)^	An ecological examination of factors influencing men’s engagement in intimate partner violence groups	Pesquisa longitudinal (retrospectiva), descritiva, do tipo estudo de caso
EUA 2013^(26)^	“En el grupo tomas conciencia (in group you become aware)”: latino immigrants’ satisfaction with a culturally informed intervention for men who batter	Pesquisa longitudinal (retrospectiva), descritiva e exploratória
África do Sul 2014^(27)^	Intimate partner violence among rural South African men: Alcohol use, sexual decision-making, and partner communication	Pesquisa longitudinal (retrospectiva), descritiva e exploratória
Austrália 2015^(28)^	‘I miss my little one a lot’: how father love motivates change in men who have used violence	Estudo descritivo-exploratório
EUA 2015^(29)^	A different kind of fraternity: psychological change and group dynamics of male batterers	Pesquisa longitudinal (retrospectiva), descritiva-exploratória, comparativa por estudos de casos
Israel 2015^(30)^	Non-violent empowerment: self-help group for male batterers on recovery	Pesquisa do tipo fenomenológica, longitudinal (retrospectiva), descritiva e exploratória
Brasil 2016^(31)^	Repercussions of imprisonment for conjugal violence: discourses of men	Pesquisa longitudinal (retrospectiva), descritiva e exploratória
Reino Unido 2016^(32)^	“Time out”: a strategy for reducing men’s violence against women in relationships?	Pesquisa longitudinal (retrospectiva), descritiva e exploratória
Brasil 2017^(33)^	La masculinité au travail au sein d’un groupe réflexif pour hommes auteurs de violence contre des femmes	Pesquisa longitudinal (retrospectiva), descritiva, do tipo estudo de caso
EUA 2017^(34)^	Exploring factors that contribute to positive change in a diverse, group-based male batterer intervention program: using qualitative data to inform implementation and adaptation efforts	Pesquisa longitudinal (retrospectiva), descritiva e exploratória
Brasil 2018^(35)^	A experiência de prisão preventiva por violência conjugal: o discurso de homens	Estudo descritivo-exploratório
Brasil 2019^(36)^	Identificando políticas públicas: Defensoria Pública e homens infratores da Lei Maria da Penha	Pesquisa exploratória, observacional
EUA 2019^(37)^	Male intimate partner violence perpetrators’ perceptions of the positives and negatives of peer interactions in group batterer intervention programs	Pesquisa do tipo etnográfica, longitudinal (retrospectiva), descritiva e exploratória
EUA 2019^(38)^	Male IPV perpetrators’ perspectives on facilitation of batterer intervention program: results from a 2-year study	Pesquisa do tipo etnográfica, longitudinal (retrospectiva), descritiva e exploratória
Brasil 2020^(39)^	Produção de sentidos em um grupo reflexivo para homens autores de violência	Pesquisa observacional, descritiva, do tipo transversal
Brasil 2020^(40)^	Social technology to prevent intimate partner violence: the vid@ group in actions with men	Pesquisa descritiva, do tipo relato de experiência
EUA 2020^(41)^	The men’s group at St. Pius V: a case study of a parish-based voluntary partner abuse intervention program	Pesquisa longitudinal (retrospectiva), descritiva, do tipo estudo de caso
África do Sul 2021^(42)^	Steppingstones and creating futures: a group*-*based approach to addressing violence against women through working with men	Pesquisa longitudinal (retrospectiva), descritiva, do tipo estudo de caso

### Características dos Estudos Incluídos

A estratégia de pesquisa não passou por corte de data, obtendo-se estudos do ano 2000 a 2021, isto é, um período relativamente recente. A maioria dos estudos selecionados foi publicada em 2015^(28#x2013;30)^, 2019^(36#x2013;38)^ e 2020^(39#x2013;41)^. Dos 28 manuscritos, dez foram desenvolvidos nos Estados Unidos da América (EUA)^(16,20,21,23,26,29,34,37,38,41)^, seis no Brasil^(31,33,35,36,39,40)^, quatro no Canadá^(15,17,19,25)^, três em Israel^(18,22,30)^, dois no Reino Unido^(24,32)^, dois na África do Sul^(27,42)^ e um na Austrália^(28)^.

A maior parte dos estudos apresentou como estratégia de prevenção da VCM abordagens de nível secundário e terciário aplicadas ao homem autor de violência. Apenas dois estudos^(21,42)^ ampliaram a prevenção para o nível primário, visando a prevenção da incidência, incluindo homens voluntários, não necessariamente autores de violência e/ou encaminhados judicialmente. Dois estudos^(31,35)^ apresentaram abordagem de prevenção exclusivamente terciária, representadas pela prisão preventiva. No Quadro 3, são apresentados os estudos selecionados com o respectivo nível de prevenção destacado nas abordagens identificadas.

**Quadro 3:** Apresentação dos estudos conforme as abordagens em nível de prevenção primária, secundária e/ou terciária – Florianópolis, SC, Brasil, 2025.

Abordagem em nível de prevenção	Estudos
Prevenção Primária	(21), (42)
Prevenção Secundária	(15), (16), (17), (18), (19), (20), (21), (22), (23), (24), (25), (26), (27), (28), (29), (30), (32), (33), (34), (36), (37), (38), (39), (40), (41), (42)
Prevenção Terciária	(15), (16), (17), (18), (19), (20), (21), (22), (23), (24), (25), (26), (27), (28), (29), (30), (32), (33), (34), (36), (37), (38), (39), (40), (41), (42)

### Abordagens de Prevenção Primária, Secundária e Terciária Aplicadas aos Homens Autores de Violência Contra Mulheres

As abordagens, majoritariamente, foram realizadas em grupo^(15#x2013;23,25–30,33,34,36–42)^. Uma intervenção ocorreu exclusivamente na modalidade de terapia individual^(24)^, e outras três mesclaram as duas abordagens anteriores^(18,22,28)^. Dois estudos descreveram a intervenção de prisão preventiva^(31,35)^ e um o ensino de uma técnica para evitar conflitos conjugais^(32)^. Nas abordagens grupais, o número de sessões variou, sendo o mínimo de oito sessões semanais^(17)^ e o máximo de 52 sessões semanais^(23,34)^. Foram prevalentes as ações com 16 sessões^(18,33,37,38)^ e 24 sessões semanais^(19,37,38)^. A duração por encontro foi de duas horas na maior parte dos estudos descritos^(16,17,19,26,29,37–39,41)^. As intervenções utilizaram-se de referenciais como as teorias feministas^(15,25,26)^ e de gênero^(22,23,27,39,42)^, terapia cognitiva-comportamental^(20,22,24–26)^, abordagem de Paulo Freire^(26,33,40,42)^, e modelo Duluth^(20,29)^.

Dos estudos que descreveram a forma de adesão dos homens, sete^(15,22,29,33,34,37,38)^ foram tanto por meio de encaminhamento judicial quanto voluntariamente, três^(17,36,39)^ apenas judicialmente e outros três^(19,21,41)^ abertos ao público de forma voluntária. Quanto ao número de homens envolvidos nos estudos e nas intervenções, o mínimo foi três^(16)^ e o máximo 177^(32)^, sendo este último realizado também com as parceiras dos homens. Entre as temáticas abordadas nas intervenções, destacaram-se: parentalidade^(18,20,21,23,25,28,34,39,41)^, impactos da violência nas crianças e famílias^(18,22,23,28,41)^, comunicação e diálogo na resolução de conflitos^(16,21,22,39,41)^, controle da raiva^(16,22,39)^, concepção e contracepção^(27,28,42)^, expectativas^(28,39,42)^ e igualdade de gênero^(23,27)^, empatia^(16,28)^, e abuso de álcool e outras drogas^(23,39)^. Em alguns casos, foram realizados encaminhamentos para outros serviços, como os Alcoólicos Anônimos^(19,35)^ e dispositivos de saúde mental^(34)^.

### Dinâmica Interdisciplinar/Interprofissional Quando na Atuação de Equipes e/Ou Serviços na Abordagem da Prevenção da Violência

Poucos estudos^(19,25,38,39)^ descreveram a dinâmica interdisciplinar/interprofissional quando na atuação de equipes e/ou serviços na abordagem de prevenção da VCM. Desses, três eram compostos por grupos mistos, com facilitadores do sexo feminino e masculino^(19,25,38)^, um apenas por homens^(33)^ e um apenas por mulheres^(39)^. A formação das pessoas facilitadoras envolveu áreas como psicologia, assistência social e demais campos das ciências sociais^(25,33,39)^. Um estudo buscou compreender a percepção dos homens sobre os facilitadores do programa de intervenção em grupo do qual participaram, de modo que, inicialmente desafiadora, a facilitação por mulheres foi importante para não tornar o grupo unilateral, além de favorecer a expressão dos sentimentos masculinos e o testemunho de interações positivas entre homens e mulheres, reconhecendo, portanto, a importância de grupos mistos. Ademais, o não julgamento por parte dos facilitadores auxiliou na mobilização para o processo de mudanças^(38)^.

### Repercussões das Abordagens aos Homens Autores de Violência e Suas Famílias

De maneira geral, os homens participantes dos estudos avaliaram positivamente^(19,20,23,24,28,36,39)^ as abordagens de prevenção da violência, evidenciando repercussões como: responsabilidade por seu comportamento^(15,18,19,21–23,32,34,37,41)^, mobilização para o processo de mudanças^(16,20,22,23,25,26,37,40,41)^, melhora na comunicação^(15,19,22,24,27,32)^ e na relação com os filhos, parceiras e família^(23,26–28,39)^, controle da raiva^(22,24,42)^, aprendizagem interpessoal por meio das histórias compartilhadas por outros homens^(30,37)^, desenvolvimento de empatia^(15,19)^, alívio da ansiedade ao expressar emoções em um ambiente seguro^(23,24)^, redução da ingesta de álcool e de outras substâncias^(27,42)^, controle de comportamentos abusivos^(19,26)^, diminuição da violência por parceiro íntimo^(42)^ e interrupção da violência física^(32)^. Um dos temas mais elencados pelas ações e que gerou maior comoção foi a respeito da parentalidade^(18,20,21,23,25,28,34,39,41)^. A temática da parentalidade esteve associada tanto ao desejo de proporcionar um ambiente familiar mais saudável para os filhos quanto de não repetir o mesmo modelo parental recebido pelo pai do homem autor de violência^(25,28,34)^.

Dois estudos apontaram que não houve reflexão por parte dos homens participantes^(31,35)^. Destaca-se que ambos tratavam da prisão preventiva, em que a raiva e o sentimento de injustiça prevaleceram entre eles. Também foi relatado sentimento de estigma social por parte dos homens ao pertencerem a uma população violenta^(22,31)^ e sintomatologia psicoemocional, como cefaleia, hipertensão, fobia e depressão^(31)^. Um estudo relatou a intenção dos participantes em utilizar a ação de prevenção como uma forma de favorecer seu julgamento^(33)^. Ainda, foram descritos casos de reincidência, um no estudo desenvolvido em 2003, no Canadá, em que um caso de violência foi relatado entre os sete ex-participantes do grupo^(17)^, e outro, realizado em 2016, nos EUA, em que um dos 48 homens participantes causou um episódio de violência enquanto participava do grupo^(29)^.

Alguns estudos também realizaram entrevistas com as esposas e ex-esposas dos homens, além de alguns familiares^(17#x2013;19,21,25,32,36)^, evidenciando a insatisfação das abordagens de prevenção por parte de algumas mulheres^(18,32,36)^. Especificamente o ensino da técnica de “dar um tempo”, ou seja, retirar-se por um período de uma situação conflituosa, apresentou potencial de melhorar a comunicação, porém também ocorreu seu uso inapropriadamente, como uma nova estratégia de controle do homem sobre a mulher. Em algumas situações, as mulheres tiveram que insistir para que o homem desse um tempo, de modo que elas se viam obrigadas a se ausentar ou, ainda, os homens saíam de cena, mas não refletiam sobre o ocorrido^(32)^.

Os dois estudos^(21,42)^ que ampliaram suas estratégias de prevenção para o nível primário evidenciaram alívio emocional pelo reconhecimento e compartilhamento de um processo de socialização masculina amplo que, muitas vezes, limita os espaços de acolhimento dos sentimentos dos homens, minando as possibilidades de reflexão. Assim, essas estratégias tiveram a capacidade de reduzir a vergonha e fornecer técnicas não violentas para lidar com a raiva^(42)^. Além disso, a inclusão de colegas, amigos e familiares desses homens foi identificada como uma intervenção importante para a responsabilização, encorajando mudanças mútuas e sistêmicas, tais como a participação ativa como agentes de transformação comunitária^(21,42)^.

Por fim, o Quadro 4 evidencia a síntese dos achados de acordo com o Score ConQual. Essa análise resultou em sete sínteses, em que o Score ConQual variou entre alto, moderado e baixo. A classificação forneceu um grau de confiança moderado para a descoberta sintetizada devido a problemas de confiabilidade (falta de esclarecimento acerca dos métodos utilizados na coleta e análise dos dados). Em quatro sínteses a credibilidade foi avaliada como moderada, uma vez que as descobertas foram classificadas como inequívocas e credíveis, isto é, nem todas representaram significativamente o achado apresentado nos estudos primários.

**Quadro 4:** Síntese dos achados e Score ConQual – Florianópolis, SC, Brasil, 2025.

Título: Abordagens de prevenção voltadas aos homens autores de violência contra mulheres			
População: homens autores de violência contra mulheres Conceito: abordagens de prevenção primária, secundária e terciária Contexto: violência contra mulheres			
**Síntese dos achados**	**Confiabilidade**	**Credibilidade**	**Score ConQual**
Repercussões positivas das abordagens aos homens, como mudanças em seu comportamento, comunicação e relacionamentos^(15,16,18-28,30,32-34,36,37,39-42)^	Alta	Total: 23 (10I + 13C)	Moderado
Ausência de reflexão por parte dos homens, especialmente em contextos de prisão preventiva^(31,35)^	Alta	Total: 2 (2I)	Alto
Relatos de reincidência de atos violentos no decorrer da participação dos homens nas abordagens^(17,29)^	Moderada	Total: 2 (2C)	Baixo
Insatisfação com as abordagens de prevenção por parte das companheiras dos homens^(18,32,36)^	Alta	Total: 3 (3I)	Alto
Abordagens com referencial nas teorias feministas e de gênero^(15,22,23,25-27,39,42)^	Alta	Total: 8 (4I + 4C)	Moderado
Importância de equipes mistas e do não julgamento por parte dos facilitadores das abordagens grupais^(19,25,38)^	Alta	Total: 3 (1I + 2C)	Moderado
Abordagens de prevenção primária apresentaram potencial de alívio emocional, responsabilização e transformação comunitária, especialmente quando da inclusão de colegas, amigos e familiares dos homens^(21,42)^	Alta	Total: 2 (1I + 1C)	Moderado

## DISCUSSÃO

As ações de prevenção primária objetivam modificar crenças sociais e envolvem ações como campanhas antiviolência e programas de empoderamento, reduzindo fatores de risco e incorporando estratégias de promoção da saúde e educação universal para o público de modo geral. O nível secundário, por sua vez, implementado quando uma intervenção primária falha ou não ocorre, busca prevenir a recorrência da violência por meio de programas de rastreio ou encaminhamentos para serviços judiciais, por exemplo. Por fim, a prevenção terciária corresponde a processos de ajustamento para atenuar problemas resultantes da violência por meio de apoio à saúde física e mental, além do cuidado social e jurídico^(1,43)^. O processo de mudança de normas sociais profundamente enraizadas é moroso e requer intervenções do nível secundário e terciário. Todavia, considerando a alta carga global de VCM e a saturação de certos recursos secundários e terciários, tais como de saúde mental, jurídico e habitacional, a ampliação da prevenção primária é crucial para a redução da VCM em longo prazo^(1,44)^.

Nos últimos anos, diversos países têm apostado no envolvimento de homens e meninos como estratégia de prevenção primária da VCM^(45)^. Acredita-se que os esforços para prevenir a VCM devem abordar os homens, tendo em vista que a masculinidade desempenha um papel crucial na formação da violência, de modo que, em grande parte, são os homens os seus autores e, portanto, possuem potencialidade de desempenhar um papel ativo na cessação e prevenção da violência^(6,46)^. Logo, abordar as normas masculinas hegemônicas é parte importante da prevenção da violência de gênero, considerando que tal masculinidade não é inata, mas construída e, portanto, está sujeita a mudanças^(47)^.

A masculinidade hegemônica apresenta menor probabilidade de apoio à equidade de gênero e maior chance de envolvimento na perpetração da VCM, enquanto ter consciência do privilégio masculino e apoiar a equidade de gênero está associado a maior disposição de se envolver em comportamentos preventivos de violência^(47)^. Desse modo, envolver os homens na prevenção da violência pode repercutir positivamente não apenas na vida de mulheres e meninas, mas na vida de homens e meninos, libertando-os de aspectos nocivos e rígidos das masculinidades tradicionais^(6,46)^. Todavia, frequentemente, o medo em ser rotulado como perpetrador e as próprias normas masculinas hegemônicas impedem os homens de discutir seus sentimentos. Além disso, a modificação e a interrupção de benefícios conferidos pelas relações desiguais de poder e gênero gera resistência às mudanças por parte dos homens^(46)^.

O tema parentalidade, como um dos mais elencados nas abordagens descritas nesta revisão, gerou comoção, reflexão e motivação para mudança. Dado que sofrer violência é frequentemente citado como um fator que contribui para a probabilidade de perpetrar violência, não raras as vezes, os homens autores de violência foram crianças que presenciaram e/ou vivenciaram situações familiares de violência^(46,48)^. As crianças que testemunham situações de violência experimentam efeitos psicossociais, emocionais e cognitivos, em termos de memória e aprendizagem, e podem reproduzir comportamentos negativos. Esse entendimento pelos homens participantes das intervenções pode gerar uma motivação autêntica ao perceberem o prejuízo e rompimento da relação com seus filhos e parceiras. Tal motivação interna possui maior potencialidade de adesão para mudança quando comparada a motivações ditas extrínsecas, como as por imposição da lei e ordens judiciais, as quais, muitas vezes, não geram a mesma reflexão^(49)^.

A presente revisão evidenciou abordagens de prevenção terciária, representadas pela prisão preventiva. No entanto, verificou-se que essas intervenções não geraram reflexão, pelo contrário. A raiva e a sensação de injustiça também esteve presente em um estudo realizado com homens que vivenciaram a prisão preventiva^(50)^. Em relação à reincidência, um estudo realizado em um grupo reflexivo para homens autores de violência no Brasil verificou que 19,7% eram reincidentes antes de participar da intervenção. Após a participação, apenas 1,3% dos participantes voltaram a ser processados pelo mesmo ato^(51)^. Todavia, entende-se que mais estudos sobre a avaliação destas ações são necessários, tendo em vista a pouca produção científica nesta área^(52)^.

Por sua vez, ações com abordagem transformadora de gênero obtiveram resultados positivos para reduzir a violência por parceiro íntimo, com redução da vitimização e perpetração em nível individual, familiar e comunitário da violência física, sexual e/ou psicológica, especialmente quando alicerçadas no contexto sociocultural da comunidade^(53)^. Por isso, são essenciais as intervenções que incentivem os homens a refletirem e a reconhecer como o gênero molda as normas, o discurso e a subjetividade em suas vidas, pensando criticamente sobre a masculinidade hegemônica^(54)^.

Além do trabalho voltado à reflexão e às atitudes equitativas de gênero, o modelo Duluth, baseado em um modelo psicoeducacional e nas teorias feministas, destaca a importância de focar no respeito, na igualdade e na responsabilização, de forma a modificar normas culturais patriarcais e desiguais na sociedade^(55)^. Ao contrário das intervenções com foco na reeducação, recuperação e/ou ressocialização, que se encaixam em uma perspectiva vertical de aprendizado, a atuação com homens autores de violência em processos grupais, reflexivos e orientados à responsabilização possui como propósito a autocompreensão e a autocrítica de sua história pessoal, de modo a viabilizar, mediante o espaço grupal e dialógico, construções coletivas para a reelaboração do modelo de masculinidade vigente^(56)^. O estabelecimento da facilitação mista, ou seja, com homens e mulheres, também pode propiciar a reflexão e atitudes equitativas de gênero por parte dos homens autores de violência, ao permitir a escuta e a perspectiva da mulher facilitadora que, de certa forma, representa sua (ex)companheira^(46,57)^.

Apesar de o trabalho com homens na prevenção da violência estar em crescimento, as ações que abordam o público masculino para além de objetos de prevenção, mas também como agentes de prevenção e de mudança social, ainda são escassas. Entende-se que as intervenções para os homens autores de VCM são fundamentais, todavia precisam estar articuladas com ações de nível primário para promoção de normas de gênero saudáveis, engajando meninos desde tenra idade até homens de uma maneira geral, incluindo autoridades, como policiais e líderes religiosos^(45,46)^. Além disso, sugere-se a ampliação de trabalhos que incluam as mulheres e demais familiares, considerando o caráter relacional da violência de gênero^(49,51)^. Apoiar e capacitar profissionais da educação, saúde e serviços sociais a questionar seus próprios preconceitos sobre estereótipos e normas de gênero; implementar educação sexual em escolas, universidades e programas comunitários; indagar sobre relacionamentos íntimos e fornecer educação sobre relacionamentos seguros e saudáveis; empoderar homens e mulheres para reconhecer e prevenir a violência nos relacionamentos; abordar a violência em grupos de cessação ao tabagismo e de redução ao uso de álcool; e promover formas positivas, não violentas e respeitosas de parentalidade em grupos de pais durante os cuidados pré e/ou pós-natais são alguns exemplos de intervenções de prevenção primária da VCM e de estratégias para promoção da saúde^(1,47,49)^.

Apesar da potencialidade em envolver os homens na prevenção da violência, há uma série de desafios a serem superados, tais como recursos humanos, fundamentação teórica e vontade política^(46)^. Profissionais da saúde têm relatado dificuldade e despreparo para o atendimento dessas situações, especialmente nos casos de violência psicológica, em que os sinais não são tão facilmente identificáveis quanto na violência física, por exemplo^(5)^. Por isso, faz-se essencial a incorporação da perspectiva interseccional e de gênero na formação profissional para abordagem e superação da violência, bem como a atuação multi e interdisciplinar^(58)^.

Como limitação, cita-se a lacuna de descrição da dinâmica interdisciplinar/interprofissional quando da atuação de equipes e/ou serviços na abordagem de prevenção da violência, o que evidencia a necessidade de pesquisas futuras nesse sentido. Espera-se que as informações e reflexões apresentadas nesta revisão de escopo sejam levadas em consideração por pesquisadores, principalmente durante a construção metodológica de estudos posteriores, assim como por profissionais envolvidos nas práticas de saúde e formuladores de políticas públicas, de modo a fortalecer e propor recomendações para melhor intervir na prevenção da violência e na promoção de relações conjugais e familiares livres de violência.

## CONCLUSÃO

A partir da presente revisão, foram mapeadas as abordagens de prevenção primária, secundária e terciária aplicadas aos homens autores de violência contra mulheres. Assim, verificou- se que a maior parte dos estudos apresentou abordagens de nível secundário e terciário aplicadas ao homem autor de violência, sendo que apenas dois estudos se enquadraram para o nível primário de prevenção.

As intervenções, de modo geral, seguiram a abordagem grupal, com potencialidade para serem replicadas em outros serviços, inclusive de saúde e para outros públicos, desde que possuam aporte teórico e humano para tal, ampliando as ações para o nível primário de prevenção, ainda incipientes. Apenas cinco estudos descreveram a dinâmica interdisciplinar, de forma que se identificou o uso de grupos facilitados por homens e mulheres, majoritariamente das áreas da psicologia, assistência social e demais campos das ciências sociais.

Na realidade brasileira, as intervenções grupais, amplamente utilizadas, conforme visualizado por esta revisão, ganham destaque na Atenção Primária à Saúde, a qual possui potencialidade de incluir homens e mulheres e abordar, de forma interdisciplinar, temas sensíveis, como a violência, a parentalidade e o abuso de álcool, por exemplo, favorecendo ações de prevenção da violência, especialmente as de nível primário.

## Data Availability

O conjunto de dados que dá suporte aos resultados deste estudo não está disponível publicamente, todavia, todo o conjunto de dados que dá suporte aos resultados deste estudo está disponível mediante solicitação à autora correspondente.

# Translation: Enfoques de prevención dirigidos a los hombres autores de violencia contra las mujeres: revisión de alcance

**DOI:** 10.1590/1980-220X-REEUSP-2025-0036es

**Authors:** Victoria Leslyê Rocha Gutmann, Gisele Cristina Manfrini, Tereza Maria Mendes Diniz de Andrade Barroso, Camila Daiane Silva, Sheila Rubia Lindner, Sandra Mara Corrêa

**Affiliations:** 1 Universidade Federal de Santa Catarina, Programa de Pós-Graduação em Enfermagem, Florianópolis, SC, Brasil.; 2 Universidade Federal de Santa Catarina, Departamento de Enfermagem, Florianópolis, SC, Brasil.; 3 Escola Superior de Enfermagem de Coimbra, Coimbra, Portugal.; 4 Universidade Federal do Rio Grande, Escola de Enfermagem, Rio Grande, RS, Brasil.; 5 Universidade Federal de Santa Catarina, Departamento de Saúde Pública, Florianópolis, SC, Brasil.

**Keywords:** Hombres, Prevención Primaria, Prevención Secundaria, Prevención Terciaria, Violencia contra la Mujer

## Abstract

**Objetivo:**

Mapear los enfoques de prevención primaria, secundaria y terciaria aplicados a los hombres autores de violencia contra las mujeres.

**Método:**

Revisión cualitativa de alcance, según las directrices del JBI. La búsqueda se realizó entre julio y octubre de 2022 en las bases de datos MEDLINE, Embase, CINAHL, PsycInfo, Scopus, Web of Science, LILACS, BDENF, IndexPsi, SciELO y Google Scholar, sin restricciones en cuanto a área geográfica, fecha de publicación y/o idioma. Todos los estudios fueron evaluados en cuanto a su fiabilidad y credibilidad, según la puntuación ConQual.

**Resultados:**

Se seleccionaron 28 estudios, la mayoría con un enfoque de nivel secundario y terciario. Solo dos estudios se enmarcaron en la prevención primaria. Las intervenciones se realizaron, en su mayoría, en grupo. La dinámica interdisciplinaria/interprofesional, raramente descrita, involucró áreas como la psicología, la asistencia social y otros campos de las ciencias sociales.

**Conclusión:**

Las intervenciones, en general, siguieron el enfoque grupal, con potencial para ser replicadas en otros servicios, incluidos los de salud y para otros públicos, siempre que cuenten con el aporte teórico y humano necesario, ampliando las acciones al nivel de prevención primaria.

## INTRODUCCIÓN

La violencia contra las mujeres (VCM) implica una evolución compleja que trasciende diferentes etapas de la vida, convirtiéndose en un problema social y global, visible a diario en datos significativos de mujeres que sufren y mueren por ser mujeres^(1)^. A nivel mundial, alrededor del 30% de las niñas y mujeres de entre 15 y 49 años afirmaron haber sufrido, al menos una vez, violencia física y/o sexual por parte de su pareja íntima, con mayor prevalencia en los países de bajos ingresos. En Brasil, esta tasa es del 23%^(2)^. Solo en 2022, el país registró 51 407 casos de violencia física. El lugar más común en el que se produjo la violencia contra la mujer fue el domicilio, en más del 80% de los casos, y los hombres fueron los principales autores en el 86,6% de los registros. En cuanto a los casos de feminicidios, se estima una tasa de 3,5 casos por cada 100 000 mujeres, en los que, una vez más, los hombres, en concreto los novios y cónyuges, fueron los principales autores^(3)^.

Los daños de la violencia, cuando no son mortales, van desde los físicos, como lesiones y heridas, hasta los psicológicos, que implican un compromiso psicoemocional de las mujeres, como baja autoestima y trastornos mentales comunes, como depresión, ansiedad, trastornos del sueño, estrés postraumático e ideación suicida, además de hiperactividad, dificultad para prestar atención y aprender, patrones alimentarios inadecuados y uso y/o abuso de drogas y medicamentos^(4)^. Estas repercusiones también afectan a la familia y a las personas cercanas a las mujeres en situación de violencia, además de a la sociedad en su conjunto, lo que se convierte en una demanda cada vez más presente en los servicios de salud. De este modo, la VCM constituye una preocupación a nivel mundial y un importante problema de salud pública, por lo que requiere medidas destinadas a combatirla y, especialmente, a prevenirla^(5)^.

En este sentido, los enfoques de prevención se dividen en niveles primario, secundario y terciario. La prevención primaria comprende las acciones realizadas antes de que se produzca la violencia, con el fin de prevenir la incidencia y la prevalencia de los casos, es decir, la perpetración inicial o la victimización. La prevención secundaria, por su parte, busca reducir la violencia que ya está en curso, identificando a las personas y familias en riesgo a través de la existencia de crisis situacionales desencadenantes de la violencia y, por lo tanto, se centra en una respuesta inmediata. Por último, la prevención terciaria se basa en respuestas a largo plazo, con el objetivo de ofrecer asistencia después de que se haya producido el hecho, evitando la reincidencia^(6)^. Aunque necesarias, las respuestas secundarias y terciarias, por sí solas, no son capaces de reducir la incidencia de la VCM, ni de prevenirla o intervenir totalmente. Los enfoques primarios proporcionan una perspectiva amplia y holística para abordar los problemas relacionales y familiares^(1)^.

Por lo tanto, se ha buscado ir más allá de los esfuerzos de prevención secundaria y terciaria para permitir mayores oportunidades de prevención primaria. Se cree que una estructura de relaciones saludables ofrece amplias oportunidades para la prevención de la violencia, ya sea promoviendo el vínculo entre el niño y el cuidador, fomentando las relaciones de orientación entre jóvenes y adultos u ofreciendo recursos financieros con miras a reducir el estrés económico familiar^(7)^. Este último tema, en particular, se ha asociado con la permanencia de las mujeres en situaciones de violencia debido a la dependencia económica. Esta dependencia se produce cuando la pareja es el sustento económico o ejerce el control de los ingresos familiares. Además, la precariedad del empleo y los salarios también contribuyen a aumentar la vulnerabilidad de las mujeres^(5)^.

Además de las variables individuales, psicológicas y relacionales, los hombres, en particular, son el resultado de una construcción sociocultural del género que normaliza y naturaliza el poder masculino sobre el femenino, basada en preceptos patriarcales que, a menudo, los acercan a actos considerados viriles y, por lo tanto, violentos. Este hecho se expresa en situaciones en las que el hombre, al percibir amenazada su identidad masculina, puede recurrir a la violencia^(8)^. Por lo tanto, la prevención de la violencia también debe considerar la transformación y la deconstrucción de formas cristalizadas de pensar, actuar y sentir la masculinidad hegemónica, así como su función social y afectiva en la relación con las mujeres y los hijos^(9)^. Es decir, la superación de la violencia requiere la construcción de formas alternativas de ser hombre, en busca de relaciones conyugales armoniosas, equitativas y no violentas^(10)^. Sin embargo, aún son escasos los estudios que involucran a los hombres autores de violencia, especialmente con carácter preventivo y/o orientados a medir la eficacia de los enfoques que involucran a este grupo^(11)^.

La Organización Mundial de la Salud reconoce la violencia contra las mujeres como una grave violación de los derechos humanos y un obstáculo para alcanzar los Objetivos de Desarrollo Sostenible (ODS) y, por ello, ha fomentado el trabajo y los estudios con los hombres para promover la equidad y la igualdad de género^(10)^. En cuanto a los ODS, destacan el 5 y el 16, que se refieren, respectivamente, a la igualdad de género para combatir las desigualdades, incluida la violencia contra las mujeres, y la promoción de sociedades pacíficas. En concreto, el ODS 5, que trata de la consecución de la igualdad de género y el empoderamiento de las mujeres, se caracteriza por ser un compromiso histórico para hacer frente, prevenir y eliminar todas las formas de violencia de género, teniendo en cuenta la persistencia y los efectos adversos de este problema^(12)^.

Así, para verificar el conjunto de evidencias sobre los enfoques para la prevención de la VCM, surgió la necesidad de realizar una revisión de la literatura, específicamente una revisión de alcance, con el fin de proporcionar una visión general de las acciones y estrategias que apuntan a la prevención primaria, secundaria y terciaria de la VCM, especialmente dirigidas a los hombres autores de violencia. De este modo, se objetivó: mapear los enfoques de prevención primaria, secundaria y terciaria aplicados a los hombres autores de violencia contra las mujeres.

## MÉTODO

### Diseño del Estudio

Se trata de una revisión de alcance, realizada según las directrices metodológicas del *Joana Briggs Institute* (JBI), siguiendo los siguientes pasos: identificación del tema y objetivo de la investigación; estrategia de búsqueda; selección de los estudios, de acuerdo con los criterios preestablecidos; mapeo y extracción de los datos; análisis de la evidencia; y presentación resumida de los resultados. Esta metodología busca proporcionar una visión general o un mapa de las evidencias y las posibles lagunas en el conocimiento, así como explorar la extensión de la literatura sobre un tema determinado, allanando el camino para futuras investigaciones con una orientación más específica^(13)^.

También se han tenido en cuenta las recomendaciones de los *Preferred Reporting Items for Systematic Reviews and Meta-Analyses* (PRISMA-ScR), que incluyen el uso de una *lista de verificación* para la elaboración y el informe de los pasos metodológicos del protocolo de esta revisión^(13)^. La revisión se registró en la plataforma *Open Science Framework*, con la identificación DOI: http://dx.doi.org/10.17605/OSF.IO/XGVA5.

### Criterios de Selección

Para definir la cuestión del estudio, se adoptó el acrónimo PCC, siendo P - Población = hombres autores de violencia contra las mujeres; C - Concepto = enfoques de prevención primaria, secundaria y terciaria; y C - Contexto = violencia contra las mujeres. De este modo, se formuló la siguiente pregunta: «¿Qué enfoques de prevención primaria, secundaria y terciaria se aplican a los hombres autores de violencia contra las mujeres?».

A partir de ahí, se definieron los siguientes criterios de inclusión: artículos originales de investigación, de tipo cualitativo, que trataran de los enfoques de prevención primaria, secundaria y/o terciaria dirigidos a hombres autores de violencia, de cualquier nacionalidad, raza o color. No se estableció ningún límite de área geográfica, fecha de publicación y/o idioma, excepto en el caso de las versiones exclusivamente en texto completo en mandarín. Los criterios de exclusión aplicados fueron tesis, disertaciones y artículos duplicados, incompletos y/o que no se correspondían con el tema de la investigación.

La búsqueda se realizó en 11 bases de datos indexadas y en la literatura gris, a saber: *PubMed*/*MEDLINE*, *Embase*, *CINAHL*, *PsycInfo*, *Scopus*, *Web of Science*, *LILACS*, *BDENF*, *IndexPsi*, *SciELO* y *Google Scholar*. Como complemento de la estrategia de búsqueda, se consultaron las referencias textuales de los estudios seleccionados para su lectura completa, que pudieron incluirse si cumplían los criterios de inclusión y la cuestión y el objetivo del estudio.

Cabe destacar que la sintaxis de búsqueda se adaptó a cada base de datos, de acuerdo con su formato. Las palabras contenidas en los títulos y resúmenes de los artículos relevantes y los términos de indexación utilizados para describir los artículos fueron analizados por dos autoras y una bibliotecaria, con el fin de componer la estrategia, de acuerdo con cada elemento del PCC, seleccionando un conjunto de descriptores disponibles en los Descriptores de Ciencias de la Salud (DeCS), en el *Medical Subject Headings* (MeSH), además de palabras clave, en portugués, inglés y español. La estrategia de búsqueda se elaboró mediante la combinación de los términos del DeCS, *MeSH* y palabras clave, con la aplicación de los operadores booleanos E/*AND* y OU/*OR*. Las estrategias de búsqueda y la asociación de los descriptores con los operadores booleanos, para cada base de datos, pueden consultarse en el Cuadro 1.

**Cuadro 1:** Estrategia de búsqueda en las bases de datos – Florianópolis, SC, Brasil, 2025.

Bases de datos	Estrategia de búsqueda
*Pubmed/MEDLINE*	((“Violence Against Women“ OR “Crimes against Women“ OR “Gender-Based Violence“[Mesh] OR “Gender-Based Violence“ OR “Domestic Violence“[Mesh] OR “Domestic Violence“ OR “Intimate Partner Violence“[Mesh] OR “Intimate Partner Violence“ OR “Dating Violence“ OR “Intimate Partner Abuse“) AND (“Primary Prevention“[Mesh] OR “Primary Prevention“ OR “Prevention“ OR “Preventive Health Services“[Mesh] OR “Preventive Health Services“ OR Preventive* OR “Secondary Prevention“[Mesh] OR “Secondary Prevention“ OR “Tertiary Prevention“[Mesh] OR “Tertiary Prevention“) AND (“Men“[Mesh] OR “Men“[Title] OR “Man“[Title] OR “Male“[Title]))
*Embase*	((“Violence Against Women“ OR “Crimes against Women“ OR “Gender-Based Violence“ OR “Domestic Violence“ OR “Intimate Partner Violence“ OR “Dating Violence“ OR “Intimate Partner Abuse“) AND (“Primary Prevention“ OR “Prevention“ OR “Preventive Health Services“ OR Preventive* OR “Secondary Prevention“ OR “Tertiary Prevention“) AND (“Men“/de OR “Men“:ti OR “Man“:ti OR “Male“:ti))
*CINAHL* *PsycInfo* *Scopus*	((“Violence Against Women“ OR “Crimes against Women“ OR “Gender-Based Violence“ OR “Domestic Violence“ OR “Intimate Partner Violence“ OR “Dating Violence“ OR “Intimate Partner Abuse“) AND (“Primary Prevention“ OR “Prevention“ OR “Preventive Health Services“ OR Preventive* OR “Secondary Prevention“ OR “Tertiary Prevention“) AND (“Men“ OR “Men“ OR “Man“ OR “Male“))
*Web of Science*	NOFT((“Violence Against Women“ OR “Crimes against Women“ OR “Gender-Based Violence“ OR “Domestic Violence“ OR “Intimate Partner Violence“ OR “Dating Violence“ OR “Intimate Partner Abuse“) AND (“Primary Prevention“ OR “Prevention“ OR “Preventive Health Services“ OR Preventive* OR “Secondary Prevention“ OR “Tertiary Prevention“) AND (“Men“ OR “Men“ OR “Man“ OR “Male“))
*LILACS* *BDENF* *IndexPsi* *SciELO*	((“Violência contra a Mulher“ OR “Crimes contra a Mulher“ OR “Crimes contra as Mulheres“ OR “Violência contra as Mulheres“ OR “Violência de Gênero“ OR “Violência Doméstica“ OR “Violência por Parceiro Íntimo“ OR “Violencia contra la Mujer“ OR “Crímenes contra la Mujer“ OR “Crímenes contra las Mujeres“ OR “Violencia contra las Mujeres“ OR “Violencia de Pareja“ OR “Violence Against Women“ OR “Crimes against Women“ OR “Gender-Based Violence“ OR “Domestic Violence“ OR “Intimate Partner Violence“ OR “Dating Violence“ OR “Intimate Partner Abuse“) AND (“Prevenção Primária“ OR “Prevenção“ OR “Serviços Preventivos de Saúde“ OR Preventiv* OR “Prevención Primaria“ OR “Prevención“ OR “Servicios Preventivos de Salud“ OR “Primary Prevention“ OR “Prevention“ OR “Preventive Health Services“ OR “Prevenção Secundária“ OR “Prevenção Terciária“ OR “Prevención Secundaria“ OR “Prevención Terciaria“ OR “Secondary Prevention“ OR “Tertiary Prevention“) AND (“Homens“ OR “Homem“ OR “masculino“ OR “Hombres“ OR “Hombre“ OR “Men“ OR “Man“ OR “Male“)
*Google Scholar*	((“Violência contra a Mulher“ OR “Violência de Gênero“ OR “Violência Doméstica“ OR “Violence Against Women“ OR “Gender-Based Violence“ OR “Domestic Violence“) AND (Prevenção OR Prevention) AND (“Homens“ OR “Homem“ OR “Men“ OR “Man“))

### Recolección de Datos

La recolección de datos se realizó entre julio y octubre de 2022, de modo que todos los estudios recuperados en las bases de datos y los cien primeros de la literatura gris se exportaron al gestor de referencias *EndNote Web*®, versión gratuita, en el que se excluyeron los resultados duplicados. A continuación, para verificar la correspondencia de los artículos con la pregunta de investigación, se procedió a la lectura de los títulos, resúmenes y descriptores en la plataforma *Rayyan Web*®.

Se siguió el método doble ciego y la inclusión o exclusión se realizó inicialmente por el título, seguido del resumen y, por último, de la lectura íntegra del texto. Tras la confirmación de la inclusión del artículo, los revisores también realizaron una búsqueda de estudios potencialmente útiles en las referencias de estos. En caso de divergencia, se previó la posibilidad de consultar a un tercer autor, lo que no fue necesario durante el proceso. Los resultados de la búsqueda y del proceso de identificación y selección de los estudios, así como los motivos de exclusión, se registraron y se recogieron en el diagrama de flujo PRISMA-ScR, según se muestra en la Figura 1.

**Figura 1:** Flujograma del proceso de selección de los artículos incluidos.

### Análisis y Tratamiento de los Datos

A partir de la lectura completa de los artículos incluidos, los revisores destacaron y resumieron la información que respondía a la pregunta y al objetivo de la revisión. Así, tras el análisis de los estudios en su totalidad, se catalogaron utilizando el *software Microsoft Excel*® y se extrajeron los datos para elaborar tablas elaboradas por los propios autores. Las variables recolectadas fueron título, autores, año de publicación, ubicación geográfica del estudio, diseño metodológico, detalles de la población, del contexto y del fenómeno de interés, incluyendo la dinámica interdisciplinaria/interprofesional en la actuación de equipos y/o servicios en el enfoque de la prevención de la violencia.

Los datos se analizaron cualitativamente, más precisamente en cuanto a su relevancia y pertinencia para el tema en estudio, destacando los aspectos metodológicos, las limitaciones, las fortalezas, las comparaciones con otros estudios en la literatura y las recomendaciones para futuras investigaciones, de acuerdo con la síntesis narrativa de los principales hallazgos. Además, como forma de evaluar la fiabilidad de los hallazgos cualitativos sintetizados, los autores siguieron las recomendaciones del enfoque ConQual. En ConQual, cada estudio cualitativo recibe una calificación de «alta», en una escala que varía entre alta, moderada, baja y muy baja. A partir de este punto de partida, cada artículo se clasifica en función de su fiabilidad y, a continuación, de su credibilidad. Mediante esta herramienta, los hallazgos de los estudios individuales pueden rebajarse o mantenerse en la clasificación inicial^(14)^.

En este sentido, la fiabilidad se establece mediante la aplicación de las cinco primeras preguntas del instrumento de evaluación crítica para estudios cualitativos, relacionadas con la adecuación de la realización de la investigación a sus objetivos y propósitos: (1) ¿Existe congruencia entre la metodología de la investigación y la cuestión o los objetivos de la investigación? (2) ¿Existe congruencia entre la metodología de la investigación y los métodos utilizados para recopilar datos? (3) ¿Existe congruencia entre la metodología de la investigación y la representación y el análisis de los datos? (4) ¿Existe una declaración que sitúe al investigador cultural o teóricamente? (5) ¿Se aborda la influencia del investigador en la investigación y viceversa^(13,14)^. Según el número de respuestas «sí» a las preguntas, se define la clasificación por artículo: de 4 a 5 respuestas «sí»: la clasificación permanece sin cambios; de 2 a 3 respuestas «sí»: la clasificación se reduce en un nivel; de 0 a 1 respuestas «sí»: la clasificación se reduce en dos niveles^(13)^.

La credibilidad, por su parte, se puntúa mediante la evaluación de la combinación de los hallazgos incluidos en las categorías asociadas. De este modo, la credibilidad se divide en: inequívoca (I): presencia de hallazgos que son, de hecho, directamente relatados/observados; creíble (C): evidencias plausibles a la luz de los datos y la estructura teórica, pero al tratarse de hallazgos interpretativos, pueden ser cuestionados; o No soportada (NS): presencia de hallazgos que no están respaldados por los datos o cuando no se aplica una clasificación inequívoca y creíble. El proceso de clasificación se define para cada hallazgo sintetizado, de la siguiente manera: si todos los hallazgos son inequívocos, la clasificación permanece sin cambios; si hay una combinación de hallazgos inequívocos/creíbles, la clasificación se reduce en un nivel; y si los hallazgos son creíbles/no respaldados, la clasificación se reduce en tres niveles^(13,14)^.

### Aspectos Éticos

Por tratarse de un estudio que utilizó información bibliográfica, no fue necesaria la aprobación del Comité de Ética en Investigación con Seres Humanos.

## RESULTADOS

La búsqueda inicial en bases de datos resultó en un total de 3.970 estudios, de los cuales 776 fueron encontrados en PubMed/MEDLINE, 546 en Embase, 613 en CINAHL, 751 en PsycInfo, 536 en Scopus, 509 en Web of Science, 34 en LILACS, siete en BDENF, cuatro en IndexPsi, 94 en SciELO y los primeros cien en Google Scholar. Tras eliminar duplicados y aplicar los criterios de inclusión y exclusión, 1.885 estudios pasaron a la lectura de títulos y resúmenes. Después de esta etapa, 35 fueron preseleccionados para lectura completa. De ellos, diez fueron excluidos y otros tres se incluyeron tras la lectura de las referencias, conformando la muestra final de 28 artículos de esta revisión. En la Cuadro 2, se presentan los artículos seleccionados en orden ascendente por año de publicación, así como una descripción del país de origen, título y diseño metodológico.

**Cuadro 2:** Características de los estudios incluidos en la revisión exploratória – Florianópolis, SC, Brasil, 2025.

País/año	Título	Diseño metodológico
Canadá 2000^(15)^	Change among batterers examining men's success stories	Investigación longitudinal (retrospectiva), descriptiva y exploratoria
EUA 2002^(16)^	Group therapy intervention for male batterers: a microethnographic study	Investigación etnográfica, observacional
Canadá 2003^(17)^	The men's peace project: a working model with male violence	Estudio longitudinal (retrospectivo), descriptivo, tipo informe de caso
Israel 2003^(18)^	Beit Noam: residential program for violent men	Estudio longitudinal (retrospectivo), descriptivo, tipo informe de caso
Canadá 2004^(19)^	A group for men who abuse their partners: participant perceptions of what was helpful	Estudio descriptivo-exploratorio
EUA 2006^(20)^	How batterer intervention programs work: participant and facilitator accounts of processes of change	Investigación longitudinal (retrospectiva), descriptiva y exploratoria
EUA 2008^(21)^	Deconstructing male violence against women the men stopping violence community-accountability model	Estudio longitudinal (retrospectivo), descriptivo, tipo informe de caso
Israel 2009^(22)^	Control of the self: partner-violent men’s experience of therapy	Investigación do tipo fenomenológica, longitudinal (retrospectiva), descriptiva y exploratoria
EUA 2010^(23)^	Culturally specific treatment for partner-abusive latino men: a qualitative study to identify and implement program components	Investigación longitudinal (retrospectiva), descriptiva y exploratoria
Reino Unido 2010^(24)^	A qualitative review of perception of change for male perpetrators of domestic abuse following abuser schema therapy (AST)	Investigación longitudinal (retrospectiva), descriptiva y exploratoria
Canadá 2012^(25)^	An ecological examination of factors influencing men’s engagement in intimate partner violence groups	Estudio longitudinal (retrospectivo), descriptivo, tipo informe de caso
EUA 2013^(26)^	“En el grupo tomas conciencia (in group you become aware)”: latino immigrants’ satisfaction with a culturally informed intervention for men who batter	Investigación longitudinal (retrospectiva), descriptiva y exploratoria
África del Sur 2014^(27)^	Intimate partner violence among rural South African men: Alcohol use, sexual decision-making, and partner communication	Investigación longitudinal (retrospectiva), descriptiva y exploratoria
Australia 2015^(28)^	‘I miss my little one a lot’: how father love motivates change in men who have used violence	Estudio descriptivo - exploratorio
EUA 2015^(29)^	A different kind of fraternity: psychological change and group dynamics of male batterers	Investigación longitudinal (retrospectiva), descriptiva - exploratoria, comparativa por estudios de casos
Israel 2015^(30)^	Non-violent empowerment: self-help group for male batterers on recovery	Investigación do tipo fenomenológica, longitudinal (retrospectiva), descriptiva y exploratoria
Brasil 2016^(31)^	Repercussions of imprisonment for conjugal violence: discourses of men	Investigación longitudinal (retrospectiva), descriptiva y exploratoria
Reino Unido 2016^(32)^	“Time out”: a strategy for reducing men’s violence against women in relationships?	Investigación longitudinal (retrospectiva), descriptiva y exploratoria
Brasil 2017^(33)^	La masculinité au travail au sein d’un groupe réflexif pour hommes auteurs de violence contre des femmes	Estudio longitudinal (retrospectivo), descriptivo, tipo informe de caso
EUA 2017^(34)^	Exploring factors that contribute to positive change in a diverse, group-based male batterer intervention program: using qualitative data to inform implementation and adaptation efforts	Investigación longitudinal (retrospectiva), descriptiva y exploratoria
Brasil 2018^(35)^	A experiência de prisão preventiva por violência conjugal: o discurso de homens	Estudio descriptivo -exploratorio
Brasil 2019^(36)^	Identificando políticas públicas: Defensoria Pública e homens infratores da Lei Maria da Penha	Investigación exploratoria, observacional
EUA 2019^(37)^	Male intimate partner violence perpetrators’ perceptions of the positives and negatives of peer interactions in group batterer intervention programs	Investigación do tipo etnográfica, longitudinal (retrospectiva), descriptiva y exploratoria
EUA 2019^(38)^	Male IPV perpetrators’ perspectives on facilitation of batterer intervention program: results from a 2-year study	Investigación do tipo etnográfica, longitudinal (retrospectiva), descriptiva y exploratoria
Brasil 2020^(39)^	Produção de sentidos em um grupo reflexivo para homens autores de violência	Investigación observacional, descriptiva, de tipo transversal
Brasil 2020^(40)^	Social technology to prevent intimate partner violence: the vid@ group in actions with men	Investigación descriptiva, de tipo relato de experiencia
EUA 2020^(41)^	The men’s group at St. Pius V: a case study of a parish-based voluntary partner abuse intervention program	Estudio longitudinal (retrospectivo), descriptivo, tipo informe de caso
África del Sur 2021^(42)^	Steppingstones and creating futures: a group-based approach to addressing violence against women through working with men	Estudio longitudinal (retrospectivo), descriptivo, tipo informe de caso

### Características de los Estudios Incluidos

La estrategia de búsqueda no tuvo fecha de corte, obteniéndose estudios desde el año 2000 hasta 2021, es decir, un periodo relativamente reciente. La mayoría de los estudios seleccionados fueron publicados en 2015^(28#x2013;30)^, 2019^(36#x2013;38)^ y 2020^(39–41)^. De los 28 manuscritos, diez se realizaron en los Estados Unidos de América(EE.UU.)^(16,20,21,23,26,29,34,37,38,41)^, seis en Brasil^(31,33,35,36,39,40)^, cuatro en Canadá^(15,17,19,25)^, tres en Israel^(18,22,30)^, dos en el Reino Unido^(24,32)^, dos en Sudáfrica^(27,42)^ y uno en Australia^(28)^.

La mayoría de los estudios presentaron enfoques de nivel secundario y terciario aplicados a los autores masculinos de la violencia como estrategia para prevenir la VCM. Sólo dos estudios^(21,42)^ extendieron la prevención al nivel primario, con el objetivo de prevenir la incidencia mediante la inclusión de voluntarios varones, no necesariamente autores de violencia y/o derivaciones judiciales. Dos estudios^(31,35)^ presentaron un enfoque de prevención exclusivamente terciario, representado por la detención preventiva. El Cuadro 3 muestra los estudios seleccionados con el respectivo nivel de prevención destacado en los enfoques identificados.

**Cuadro 3:** Presentación de los estudios según los abordajes de prevención primaria, secundaria y/o terciaria – Florianópolis, SC, Brasil, 2025.

Abordajes por nivel de prevención	Estudios
Prevención Primaria	(21), (42)
Prevención Secundaria	(15), (16), (17), (18), (19), (20), (21), (22), (23), (24), (25), (26), (27), (28), (29), (30), (32), (33), (34), (36), (37), (38), (39), (40), (41), (42)
Prevención Terciaria	(15), (16), (17), (18), (19), (20), (21), (22), (23), (24), (25), (26), (27), (28), (29), (30), (32), (33), (34), (36), (37), (38), (39), (40), (41), (42)

### Enfoques de Prevención Primaria, Secundaria y Terciaria Aplicados a los Hombres Autores de Actos de Violencia Contra las Mujeres

La mayoría de los enfoques se llevaron a cabo en grupo^(15–23,25–30,33,34,36–42)^. Una intervención tuvo lugar exclusivamente en forma de terapia individual^(24)^, y otras tres mezclaron los dos enfoques anteriores^(18,22,28)^. Dos estudios describieron la intervención de detención preventiva^(31,35)^ y uno la enseñanza de una técnica para evitar conflictos conyugales^(32)^. En los abordajes grupales, el número de sesiones varió, con un mínimo de ocho sesiones semanales^(17)^ y un máximo de 52 sesiones semanales^(23,34)^. Predominaron las acciones con 16 sesiones^(18,33,37,38)^ y 24 sesiones semanales^(19,37,38)^. La duración de cada reunión fue de dos horas en la mayoría de los estudios descritos^(16,17,19,26,29,37–39,41)^. Las intervenciones utilizaron referentes como las teorías feministas^(15,25,26)^ y de género^(22,23,27,39,42)^, la terapia cognitivo-conductual^(20,22,24–26)^, el enfoque de Paulo Freire^(26,33,40,42)^ y el modelo de Duluth^(20,29)^.

De los estudios que describieron la forma de ingreso de los hombres, siete^(15,22,29,33,34,37,38)^ fueron tanto por derivación judicial como de forma voluntaria, tres^(17,36,39)^ sólo judicialmente y otros tres^(19,21,41)^ abiertos al público de forma voluntaria. En cuanto al número de hombres implicados en los estudios e intervenciones, el mínimo fue de tres^(16)^ y el máximo de 177^(32)^, realizándose estas últimas también con las parejas de los hombres. Los temas tratados en las intervenciones fueron crianza^(18,20,21,23,25,28,34,39,41)^, impactos de la violencia en los hijos y las familias^(18,22,23,28,41)^, comunicación y diálogo en la resolución de conflictos^(16,21,22,39,41)^, manejo de la ira^(16,22,39)^, concepción y anticoncepción^(27,28,42)^, expectativas^(28,39,42)^ e igualdad de género^(23,27)^, empatía^(16,28)^, y abuso de alcohol y otras drogas^(23,39)^. En algunos casos, se realizaron derivaciones a otros servicios, como Alcohólicos Anónimos^(19,35)^ y centros de salud mental^(34)^.

### Dinámica Interdisciplinar/Interprofesional Cuando los Equipos Y/O Servicios Trabajan Juntos Para Prevenir la Violencia

Pocos estudios^(19,25,38,39)^ han descrito la dinámica interdisciplinar/interprofesional cuando los equipos y/o servicios trabajan juntos para prevenir la VCM. De ellos, tres consistían en grupos mixtos, con facilitadores femeninos y masculinos^(19,25,38)^, uno sólo con hombres^(33)^ y otro sólo con mujeres^(39)^. La formación de los facilitadores incluía áreas como la psicología, el trabajo social y otros campos de las ciencias sociales^(25,33,39)^. Un estudio trató de comprender las percepciones de los hombres sobre los facilitadores del programa de intervención en grupo en el que participaban y descubrió que, en un principio desafiante, la facilitación por parte de mujeres era importante para que el grupo no fuera unilateral, así como para fomentar la expresión de sentimientos masculinos y presenciar interacciones positivas entre hombres y mujeres, reconociendo así la importancia de los grupos mixtos. Además, la ausencia de prejuicios por parte de los facilitadores ayudó a movilizar el proceso de cambio^(38)^.

### Repercusiones de los Enfoques Sobre los Hombres Autores de Violencia y sus Familias

En general, los hombres que participaron en los estudios evaluaron positivamente los enfoques de prevención de la violencia^(19,20,23,24,28,36,39)^, destacando repercusiones como: la responsabilidad por su comportamiento^(15,18,19,21–23,32,34,37,41)^, la movilización para el proceso de cambio^(16,20,22,23,25,26,37,40,41)^, la mejora de la comunicación^(15,19,22,24,27,32)^ y de las relaciones con los hijos, la pareja y la familia^(23,26–28,39)^, el control de la ira^(22,24,42)^, el aprendizaje interpersonal a través de las historias compartidas por otros hombres^(30,37)^, el desarrollo de la empatía^(15,19)^, el alivio de la ansiedad mediante la expresión de emociones en un entorno seguro^(23,24)^, la reducción del consumo de alcohol y otras sustancias^(27,42)^, el control de los comportamientos abusivos^(19,26)^, la reducción de la violencia en la pareja^(42)^ y el cese de la violencia física^(32)^. Uno de los temas más frecuentemente enumerados por las acciones y que generó mayor revuelo fue la crianza de los hijos^(18,20,21,23,25,28,34,39,41)^. El tema de la crianza se asoció tanto al deseo de proporcionar un ambiente familiar más saludable para los hijos como a no repetir el mismo modelo de crianza recibido por el padre del agresor^(25,28,34)^.

Dos estudios mostraron que no hubo reflexión por parte de los participantes masculinos^(31,35)^. Cabe destacar que ambos trataban sobre la detención preventiva, en la que predominaba entre ellos la ira y la sensación de injusticia. Los hombres también informaron de sentimientos de estigma social al pertenecer a una población violenta^(22,31)^ y de síntomas psicoemocionales como dolores de cabeza, hipertensión, fobia y depresión^(31)^. Un estudio informó de la intención de los participantes de utilizar la acción de prevención como forma de mejorar su juicio^(33)^. También se han descrito casos de reincidencia, uno en un estudio realizado en 2003 en Canadá, en el que se informó de un caso de violencia entre los siete exparticipantes del grupo^(17)^, y otro, realizado en 2016 en EE.UU., en el que uno de los 48 hombres participantes provocó un episodio de violencia mientras participaba en el grupo^(29)^.

Algunos estudios también llevaron a cabo entrevistas con las esposas y ex esposas de los hombres, así como con algunos miembros de la familia^(17#x2013;19,21,25,32,36)^, mostrando que algunas mujeres estaban insatisfechas con los enfoques de prevención^(18,32,36)^. En concreto, la enseñanza de la técnica del «tiempo fuera», es decir, retirarse durante un tiempo de una situación conflictiva, tenía el potencial de mejorar la comunicación, pero también se utilizó de forma inadecuada, como una nueva estrategia de los hombres para controlar a las mujeres. En algunas situaciones, las mujeres tuvieron que insistir para que el hombre se tomara un descanso, por lo que se vieron obligadas a marcharse, o los hombres abandonaron la escena pero no reflexionaron sobre lo sucedido^(32)^.

Los dos estudios^(21,42)^ que extendieron sus estrategias de prevención al nivel primario mostraron alivio emocional al reconocer y compartir un amplio proceso de socialización masculina que a menudo limita los espacios donde se aceptan los sentimientos de los hombres, socavando las posibilidades de reflexión. Así, estas estrategias consiguieron reducir la vergüenza y proporcionar técnicas no violentas para hacer frente a la ira^(42)^. Además, la inclusión de los colegas, amigos y familiares de estos hombres se identificó como una intervención importante para la rendición de cuentas, fomentando cambios mutuos y sistémicos, como la participación activa como agentes de transformación de la comunidad^(21,42)^.

Por último, el cuadro 4 resume las conclusiones según la puntuación ConQual. Este análisis dio como resultado siete síntesis, en las que la Puntuación ConQual varió entre alta, moderada y baja. La clasificación proporcionó un grado de confianza moderado para la conclusión sintetizada debido a problemas de fiabilidad (falta de aclaración sobre los métodos utilizados para recopilar y analizar los datos). En cuatro síntesis, la credibilidad se evaluó como moderada, ya que los hallazgos se clasificaron como inequívocos y creíbles, es decir, no todos representaban significativamente el hallazgo presentado en los estudios primarios.

**Cuadro 4:** Resumen de hallazgos y puntuación ConQual – Florianópolis, SC, Brasil, 2025.

Título: Enfoques de prevención para los autores masculinos de violencia contra las mujeres			
Población: hombres autores de actos de violencia contra las mujeres Concepto: enfoques de prevención primaria, secundaria y terciaria Contexto: violencia contra las mujeres			
**Síntesis de los hallazgos**	**Confiabilidad**	**Credibilidad**	**Escore ConQual**
Repercusiones positivas de los acercamientos a los hombres, como cambios en su comportamiento, comunicación y relaciones^(15,16,18-28,30,32-34,36,37,39-42)^	Alta	Total: 23 (10I + 13C)	Moderado
Falta de reflexión por parte de los hombres, especialmente en el contexto de la detención preventiva^(31,35)^	Alta	Total: 2 (2I)	Alto
Informes de recurrencia de actos violentos durante la participación de los hombres en los enfoques^(17,29)^	Moderada	Total: 2 (2C)	Bajo
Insatisfacción con los enfoques de prevención por parte de las parejas de los hombres^(18,32,36)^	Alta	Total: 3 (3I)	Alto
Enfoques basados en teorías feministas y de género^(15,22,23,25-27,39,42)^	Alta	Total: 8 (4I + 4C)	Moderado
La importancia de los equipos mixtos y la ausencia de prejuicios por parte de los facilitadores de los enfoques de grupo^(19,25,38)^	Alta	Total: 3 (1I + 2C)	Moderado
Los enfoques de prevención primaria tienen potencial para el alivio emocional, la responsabilidad y la transformación de la comunidad, especialmente cuando incluyen a los colegas, amigos y familiares de los hombres^(21,42)^	Alta	Total: 2 (1I + 1C)	Moderado

## DISCUSIÓN

Las acciones de prevención primaria buscan cambiar las creencias sociales e implican acciones como campañas contra la violencia y programas de empoderamiento, reduciendo los factores de riesgo e incorporando estrategias de promoción de la salud y educación universal para el público en general. El nivel secundario, por su parte, implementado cuando una intervención primaria falla o no tiene lugar, busca prevenir la recurrencia de la violencia a través de programas de detección o derivaciones a servicios judiciales, por ejemplo. Por último, la prevención terciaria corresponde a los procesos de adaptación para aliviar los problemas derivados de la violencia mediante el apoyo a la salud física y mental, así como la atención social y jurídica^(1,43)^. El proceso de cambio de normas sociales profundamente arraigadas lleva tiempo y requiere intervenciones en los niveles secundario y terciario. Sin embargo, teniendo en cuenta la elevada carga mundial de la VCM y la saturación de ciertos recursos secundarios y terciarios, como los de salud mental, jurídicos y de vivienda, ampliar la prevención primaria es crucial para reducir la VCM a largo plazo^(1,44)^.

En los últimos años, varios países se han centrado en implicar a hombres y niños como estrategia de prevención primaria de la VCM^(45)^. Se cree que los esfuerzos para prevenir la VCM deben dirigirse a los hombres, dado que la masculinidad desempeña un papel crucial en la configuración de la violencia, de modo que los hombres son en gran medida los autores y, por tanto, tienen el potencial de desempeñar un papel activo para detener y prevenir la violencia^(6,46)^. Por lo tanto, abordar las normas masculinas hegemónicas es una parte importante de la prevención de la violencia de género, teniendo en cuenta que la masculinidad no es innata, sino construida y, por lo tanto, sujeta a cambios^(47)^.

La masculinidad hegemónica tiene menos probabilidades de apoyar la equidad de género y más probabilidades de participar en la perpetración de la violencia de género, mientras que ser consciente del privilegio masculino y apoyar la equidad de género se asocia con una mayor disposición a participar en comportamientos de prevención de la violencia^(47)^. De este modo, implicar a los hombres en la prevención de la violencia puede tener un impacto positivo no sólo en la vida de las mujeres y las niñas, sino también en la de los hombres y los niños, liberándolos de los aspectos nocivos y rígidos de las masculinidades tradicionales^(6,46)^. Sin embargo, el miedo a ser etiquetado como agresor y las normas masculinas hegemónicas impiden a menudo que los hombres hablen de sus sentimientos. Además, la modificación e interrupción de los beneficios conferidos por las relaciones desiguales de poder y de género genera resistencia al cambio por parte de los hombres^(46)^.

El tema de la parentalidad, como uno de los más mencionados en los enfoques descritos en esta revisión, ha generado conmoción, reflexión y motivación para el cambio. Dado que sufrir violencia se cita a menudo como un factor que contribuye a la probabilidad de perpetrar violencia, no es infrecuente que los perpetradores masculinos de violencia hayan sido niños que han presenciado y/o experimentado situaciones familiares de violencia^(46,48)^. Los niños que presencian situaciones de violencia experimentan efectos psicosociales, emocionales y cognitivos, en términos de memoria y aprendizaje, y pueden reproducir comportamientos negativos. Esta comprensión por parte de los hombres que participan en las intervenciones puede generar una auténtica motivación cuando se dan cuenta del daño y la ruptura de la relación con sus hijos y parejas. Esta motivación interna tiene un mayor potencial de adhesión al cambio en comparación con las llamadas motivaciones extrínsecas, como las impuestas por la ley o las órdenes judiciales, que a menudo no generan la misma reflexión^(49)^.

Esta revisión destacó los enfoques de prevención terciaria, representados por la detención preventiva. Sin embargo, se constató que estas intervenciones no generaban reflexión, sino todo lo contrario. La rabia y el sentimiento de injusticia también estuvieron presentes en un estudio sobre hombres que habían experimentado la detención preventiva^(50)^. En cuanto a la reincidencia, en un estudio realizado en un grupo de reflexión para hombres autores de actos violentos en Brasil se descubrió que el 19,7% eran reincidentes antes de participar en la intervención. Después de participar, sólo el 1,3% de los participantes volvieron a ser procesados por el mismo acto^(51)^. Sin embargo, se entiende que son necesarios más estudios sobre la evaluación de estas acciones, dada la falta de producción científica en esta área^(52)^.

A su vez, las acciones con enfoque transformador de género han tenido resultados positivos en la reducción de la violencia de pareja, con disminución de la victimización y perpetración de violencia física, sexual y/o psicológica a nivel individual, familiar y comunitario, especialmente cuando se basan en el contexto sociocultural de la comunidad^(53)^. Por ello, son esenciales las intervenciones que animen a los hombres a reflexionar y reconocer cómo el género configura las normas, el discurso y la subjetividad en sus vidas, reflexionando críticamente sobre la masculinidad hegemónica^(54)^.

Además del trabajo dirigido a la reflexión y a las actitudes equitativas de género, el modelo de Duluth, basado en un modelo psicoeducativo y en teorías feministas, subraya la importancia de centrarse en el respeto, la igualdad y la responsabilidad para cambiar las normas culturales patriarcales y desiguales de la sociedad^(55)^. A diferencia de las intervenciones centradas en la reeducación, recuperación y/o resocialización, que se inscriben en una perspectiva de aprendizaje vertical, el trabajo con hombres perpetradores de violencia en procesos grupales, reflexivos y orientados a la rendición de cuentas tiene como objetivo la autocomprensión y la autocrítica de su historia personal, para posibilitar, a través del espacio grupal y dialógico, construcciones colectivas para la reelaboración del actual modelo de masculinidad^(56)^. La facilitación mixta, es decir, con hombres y mujeres, también puede favorecer la reflexión y las actitudes de equidad de género por parte de los hombres autores de violencia, al permitirles escuchar la perspectiva de la facilitadora que, de alguna manera, representa a su (ex) pareja^(46,57)^.

Aunque el trabajo con hombres para prevenir la violencia va en aumento, todavía son pocas las acciones que se dirigen al público masculino más allá de ser objetos de prevención, sino también como agentes de prevención y cambio social. Se entiende que las intervenciones para los hombres perpetradores de VCM son fundamentales, pero necesitan articularse con acciones a nivel primario para promover normas de género saludables, involucrando desde niños hasta hombres en general, incluyendo autoridades como policías y líderes religiosos^(45,46)^. Además, se sugiere ampliar el trabajo para incluir a las mujeres y otros miembros de la familia, considerando la naturaleza relacional de la violencia de género^(49,51)^.

Apoyar y formar a los profesionales de la educación, la salud y los servicios sociales para que cuestionen sus propios prejuicios sobre los estereotipos y las normas de género; implantar la educación sexual en las escuelas, las universidades y los programas comunitarios; indagar sobre las relaciones íntimas y proporcionar educación sobre relaciones seguras y sanas; capacitar a hombres y mujeres para reconocer y prevenir la violencia en las relaciones; abordar la violencia en grupos para dejar de fumar y reducir el consumo de alcohol; y promover formas de crianza positivas, no violentas y respetuosas en grupos de crianza durante la atención prenatal y/o postnatal son algunos ejemplos de intervenciones de prevención primaria de la violencia contra la mujer y estrategias de promoción de la salud^(1,47,49)^.

A pesar del potencial de implicar a los hombres en la prevención de la violencia, hay una serie de retos que superar, como los recursos humanos, los fundamentos teóricos y la voluntad política^(46)^. Los profesionales de la salud han informado de la dificultad y la falta de preparación para hacer frente a estas situaciones, especialmente en los casos de violencia psicológica, donde los signos no son tan fácilmente identificables como en la violencia física, por ejemplo^(5)^. Por ello, es fundamental incorporar la perspectiva interseccional y de género en la formación profesional para el abordaje y superación de la violencia, así como la actuación multi e interdisciplinar^(58)^.

Una limitación de este estudio es la falta de descripción de las dinámicas interdisciplinares/interprofesionales cuando los equipos y/o servicios trabajan para prevenir la violencia, lo que pone de manifiesto la necesidad de futuras investigaciones en este ámbito. Se espera que las informaciones y reflexiones presentadas en esta revisión exploratoria sean tomadas en consideración por investigadores, especialmente durante la construcción metodológica de estudios posteriores, así como por profesionales involucrados en prácticas de salud y formuladores de políticas públicas, a fin de fortalecer y proponer recomendaciones para una mejor intervención en la prevención de la violencia y en la promoción de relaciones conyugales y familiares libres de violencia.

## CONCLUSIÓN

Esta revisión mapeó los abordajes de prevención primaria, secundaria y terciaria aplicados a los hombres perpetradores de violencia contra las mujeres. Se encontró que la mayoría de los estudios presentaban enfoques de nivel secundario y terciario aplicados a los agresores masculinos de violencia, y sólo dos estudios cubrían el nivel primario de prevención.

Las intervenciones generalmente siguieron un abordaje grupal, con potencial de ser replicadas en otros servicios, incluyendo los de salud y para otros públicos, siempre y cuando cuenten con el soporte teórico y humano para hacerlo, ampliando las acciones al nivel primario de prevención, aún incipiente. Sólo cinco estudios describieron dinámicas interdisciplinarias, que identificaron el uso de grupos facilitados por hombres y mujeres, en su mayoría de las áreas de psicología, trabajo social y otras ciencias sociales.

En Brasil, las intervenciones grupales son ampliamente utilizadas, como ha demostrado esta revisión, y son particularmente importantes en la Atención Primaria de Salud, que tiene el potencial de incluir a hombres y mujeres y abordar temas sensibles como la violencia, la crianza de los hijos y el abuso de alcohol de forma interdisciplinaria, favoreciendo las acciones de prevención de la violencia, especialmente en el nivel primario.

## Data Availability

El conjunto de datos que respalda los hallazgos de este estudio no está disponible públicamente, sin embargo, el conjunto de datos completo que respalda los hallazgos de este estudio está disponible a pedido del autor correspondiente.
